# Explaining the association between social and lifestyle factors and cognitive functions: a pathway analysis in the Memento cohort

**DOI:** 10.1186/s13195-022-01013-8

**Published:** 2022-05-18

**Authors:** Leslie Grasset, Cécile Proust-Lima, Jean-François Mangin, Marie-Odile Habert, Bruno Dubois, Claire Paquet, Olivier Hanon, Audrey Gabelle, Mathieu Ceccaldi, Cédric Annweiler, Renaud David, Therese Jonveaux, Catherine Belin, Adrien Julian, Isabelle Rouch-Leroyer, Jérémie Pariente, Maxime Locatelli, Marie Chupin, Geneviève Chêne, Carole Dufouil, on behalf of the Memento Cohort Study group

**Affiliations:** 1 University of Bordeaux, Inserm, Bordeaux Population Health Research Center, UMR 1219; Inserm, CIC1401-EC, F-33000 Bordeaux, France; 2 CATI Multicenter Neuroimaging Platform, 75000 Paris, France; 3 Neurospin CEA Paris Saclay University, 91190 Gif-sur-Yvette, France; 4 Sorbonne Université, CNRS, INSERM, Laboratoire d’Imagerie Biomédicale, LIB, F-75006 Paris, France; 5 AP-HP, Hôpital Pitié-Salpêtrière, Médecine Nucléaire, 75013 Paris, France; 6 IM2A AP-HP INSERM UMR-S975 Groupe Hospitalier Pitié-Salpêtrière Institut de La Mémoire Et de La Maladie d’Alzheimer, Institut du Cerveau Et de La Moelle Épinière Sorbonne, Université Paris, Paris, France; 7 Centre de Neurologie Cognitive Hôpital Lariboisière, Université de Paris, INSERMU1144, F-75010 Paris, France; 8 Geriatric Department Broca Hospital, Université Paris Descartes, Sorbonne Paris Cité, APHP, EA 4468, F-75013 Paris, France; 9 Centre Mémoire Ressources Recherche Département de Neurologie CHU Gui de Chauliac, 34000 Montpellier, France; 10 Inserm U1061, La Colombière Université de Montpellier, 34000 Montpellier, France; 11 CMMR PACA Ouest CHU Timone APHM & Aix Marseille Univ INSERM INS Inst Neurosci Syst, 13000 Marseille, France; 12 Department of Geriatric Medicine, Research Center On Autonomy and Longevity; UPRES EA 4638, Angers University HospitalAngers University Memory ClinicUniversity of Angers, 49000 Angers, France; 13 Department of Medical Biophysics, Schulich School of Medicine and Dentistry, Robarts Research Institute, the University of Western Ontario, London, ON Canada; 14 Centre Mémoire de Ressources et de Recherche (CMRR) – CHU Nice, Centre de Recherche Edmond Et Lily SAFRA, CoBTeK “Cognition Behaviour Technology” – Université Côte d’Azur, Institut Claude Pompidou, EA 7276, 06000 Nice, France; 15 Centre Mémoire de Ressources Et de Recherche de Lorraine, Laboratoire Lorrain de Psychologie Et de Neurosciences de La Dynamique Des Comportements, Unité Cognitivo Comportementale CHRU Nancy, Université de Lorraine, 2LPN EA 7489, 54000 Nancy, France; 16 Service de Neurologie Hôpital Saint-Louis AP-HP, 75010 Paris, France; 17 Service de Neurologie CHU La Milétrie Centre Mémoire de Ressources Et de Recherche, 86000 Poitiers, France; 18 Cellule Régionale d’observation de La Maladie d’Alzheimer Et Des Maladies Apparentées, CHU de Saint Etienne, 42000 Saint-Etienne, France; 19 Department of Neurology, Toulouse University Hospital, 31000 Toulouse, France; 20 Toulouse NeuroImaging Center, Universite de Toulouse, Inserm, UPS, 31000 Toulouse, France; 21 Institut du Cerveau Et de La Moelle Épinière, CNRS, Inserm, UMR 7225, U 1127, Sorbonne Université, CATI, F-75013 Paris, France; 22 Pole de Sante Publique Centre Hospitalier Universitaire (CHU) de Bordeaux, 33000 Bordeaux, France

**Keywords:** Social factors, Lifestyle factors, Cognitive function, Brain markers, Pathology, Pathways

## Abstract

**Background:**

This work aimed to investigate the potential pathways involved in the association between social and lifestyle factors, biomarkers of Alzheimer’s disease and related dementia (ADRD), and cognition.

**Methods:**

The authors studied 2323 participants from the Memento study, a French nationwide clinical cohort. Social and lifestyle factors were education level, current household incomes, physical activity, leisure activities, and social network from which two continuous latent variables were computed: an early to midlife (EML) and a latelife (LL) indicator. Brain magnetic resonance imaging (MRI), lumbar puncture, and amyloid-positron emission tomography (PET) were used to define three latent variables: neurodegeneration, small vessel disease (SVD), and AD pathology. Cognitive function was defined as the underlying factor of a latent variable with four cognitive tests. Structural equation models were used to evaluate cross-sectional pathways between social and lifestyle factors and cognition.

**Results:**

Participants’ mean age was 70.9 years old, 62% were women, 28% were apolipoprotein-ε4 carriers, and 59% had a Clinical Dementia Rating (CDR) score of 0.5. Higher early to midlife social indicator was only directly associated with better cognitive function (direct *β* = 0.364 (0.322; 0.405), with no indirect pathway through ADRD biomarkers (total *β* = 0.392 (0.351; 0.429)). In addition to a direct effect on cognition (direct *β* = 0.076 (0.033; 0.118)), the association between latelife lifestyle indicator and cognition was also mostly mediated by an indirect effect through lower neurodegeneration (indirect *β* = 0.066 (0.042; 0.090) and direct *β* =  − 0.116 (− 0.153; − 0.079)), but not through AD pathology nor SVD.

**Conclusions:**

Early to midlife social factors are directly associated with higher cognitive functions. Latelife lifestyle factors may help preserve cognitive functions through lower neurodegeneration.

**Supplementary Information:**

The online version contains supplementary material available at 10.1186/s13195-022-01013-8.

## Introduction

Due to the continuous increase in life expectancy, a growing part of the population is expected to be at risk for severe cognitive impairment and age-related disorders such as Alzheimer’s disease and related dementias (ADRD), and there is an urgent need to accelerate research on ADRD prevention [1]. Several social and lifestyle factors, such as high educational level, higher socioeconomic status (SES), and engagement in stimulating activities (either mentally, physically, or socially), have been hypothesized to promote resilience against ADRD and cognitive decline [2–8]. However, the different mechanisms involved, as well as the role of ADRD biomarkers (i.e. amyloid-β and tau (AD biomarkers), cerebrovascular pathology, and neurodegeneration) in the association between these factors and cognitive function remain unclear. In addition, previous studies have often investigated social and lifestyle factors individually at one point in time, which may not entirely capture the interconnected nature of risk and protective factors against ADRD over the life course.

Results of studies investigating the impact of social and lifestyle factors on ADRD biomarkers have been mixed. Some have reported a lower level of amyloid plaques, tauopathies, neurodegeneration, or small vessel disease (SVD) pathology among individuals with more stimulating levels of social and lifestyle factors [9–16], while others showed no association between these factors and the level or change of AD biomarkers, brain volumes, or white matter hyperintensities (WMH) [9, 10, 16–24]. Mediation analysis using comprehensive disease burden measures is needed to improve our understanding of the different pathways linking social and lifestyle factors to cognitive functions. A few previous studies using a combination of factors have reported a mediated association between more stimulating levels of social and lifestyle factors and higher cognitive functions through better cerebrovascular health and lower neurodegeneration, but not through AD biomarkers such as amyloid-beta [13, 14, 18, 25].

The objective of our study was thus to investigate the associations between combined social and lifestyle factors at two different times over the life course (i.e. early to midlife and latelife respectively), multimodal ADRD biomarkers, and cognitive functions, through mediation analysis, in Memento, a French nationwide large clinical cohort.

## Methods

### Study sample

The Memento cohort is a prospective clinic-based study aiming at better understanding the natural history of ADRD and identifying new subtypes of the disease. Details of the study have been previously published [26]. The study sample was drawn from a defined population. Briefly, 2323 participants consulting within 28 French memory clinics and presenting with either isolated cognitive complaints or recently diagnosed mild cognitive impairment (MCI) were recruited from April 2011 to June 2014. MCI was defined as [1] performing 1 SD worse than the mean of the group with the same subject’s own age, sex and education level in one or more cognitive domains, this deviation being identified for the first time through cognitive tests performed recently (less than 6 months preceding screening phase), and [2] having a Clinical Dementia Rating (CDR) ≤ 0.5 and not being demented. Participants were examined at baseline and followed every 6 to 12 months up to 5 years. Baseline data collection during the face-to-face interview included socio-demographic characteristics, lifestyle factors, neurological and physical examination, and a full neuropsychological battery. Brain MRI (mandatory), lumbar puncture (LP) (optional), and 18F-fluorodeoxyglucose positon emission tomography (FDG-PET) (optional) were performed at baseline and every 2 years. Amyloid-PET scans were obtained as part of the MEMENTO-Amyging ancillary study.

### Social and lifestyle variables

We selected a set of social and lifestyle factors available for the Memento participants and hypothesized to be cognitively stimulating and associated with cognitive functions during early to midlife and latelife [8].

Early to midlife social factors included:

- Education level defined in 4 categories: no diploma or primary school level, validated short secondary school level, validated long secondary school level, and some college or higher
- Occupations gathered by complexity level: lower (blue-collar workers: i.e. …), intermediate (white-collar workers: i.e. …), and higher complexity (executive positions: i.e. …)
- Monthly household income recorded in 8 categories ranging from 400–800€ to 6000€+

Latelife lifestyle factors included:

- Physical activity assessed with the International Physical Activity Questionnaire (IPAQ) [27], defined as low, moderate, and vigorous
- Leisure activity in 4 categories being quartiles (< 6; 6–7; 8–9; >9) according to the number of physical, cognitive, and social activities carried out at least once a week from a list of 15 (producing a score from 0 to 15)
- Social network index (SNI), adapted from The Berkman-Syme SNI, combining information on marital status, sociability (number of close relatives/friends), and membership in community organizations [28]. SNI ranged from 0 to 3, with higher scores corresponding to increasing social connectedness.

### ADRD biomarkers

ADRD biomarkers were measured through either neuroimaging (MRI, FDG-PET, and amyloid-PET) or CSF.

All neuroimaging acquisitions and analyses were coordinated by the Center for Acquisition and Treatment of Images (CATI; cati-neuroimaging.com), a platform dedicated to the management of multicentre neuroimaging [29]. Scans were harmonized across centre, centralized, quality checked, and postprocessed to obtain standardized measurements.

#### MRI measures

Brain MRI was mandatory, and 86% of participants had a 3.0 Tesla MRI scan (vs 1.5 Tesla otherwise) at baseline. Neurodegeneration and white matter lesion measures were computed using automated procedures. Whole-brain and grey/white volumetry was performed using the method “segment” in SPM12 software. Hippocampal volumetry was performed with SACHA software [30, 31]. Cortical thickness was computed with the FreeSurfer software for each region of interest (ROI) of the Desikan-Killiany Atlas,[32, 33] and the AD cortical signature was estimated (including entorhinal, inferior temporal, middle temporal, inferior parietal, fusiform, and precuneus) [34]. White matter hyperintensity volume (WMHV) was estimated using WHASA software [35] complemented by a visual assessment of deep and periventricular lesions done centrally by two trained raters using the Fazekas scale [36].

#### FDG-PET

FDG-PET scans were acquired in 57% of participants 30 min after injection of 2 MBq/kg of 2-deoxy-2-18F-fluoro-d-glucose. All acquisitions consisted of 3 × 5-min frames. Images were then reconstructed using an iterative algorithm, and last, frames were realigned, averaged, and quality-checked. Mean FDG-PET uptake for a set of disease-specific ROIs (posterior cingulate cortex, inferior parietal lobule, precuneus and inferior temporal gyrus) inferred from the ADNI database was estimated [37]. Further details on the FDG-PET procedure are available in Additional file 1.

#### Amyloid-PET

Amyloid PET examinations (MEMENTO-Amyging substudy) were performed in 28% of participants using either Florbetapir (18F) or Flutemetamol (18F) radioligands. Florbetapir scans (3 × 5 min) were acquired 50 min after injection of 370 (± 10%) MBq. Flutemetamol scans (4 × 5 min) were acquired 90 min after injection of 185 (± 10%) MBq. Images were then reconstructed using an iterative algorithm. Frames were realigned, averaged, and quality-checked. Standard uptake value ratios (SUVR) for target areas such as the medial frontal cortex, temporal cortex, parietal cortex, posterior cingulate cortex, anterior cingulate cortex, and precuneus were calculated with respect to the entire cerebellum [38]. Further details are available in Additional file 1.

#### Cerebrospinal fluid sampling AD markers

LP was performed in 18% of participants at baseline. Each CSF sample was transferred to a local biobank within 4 h after collection and was centrifuged at 1000 × *g* at 4 °C for 10 min. CSF samples were aliquoted in polypropylene tubes (16 tubes of 250 μl) and stored at − 80 °C. All tubes were further shipped for storage in a centralized biobank (LAG-CRB, Pasteur Institut Lille, BB-0033–00,071). Measurements from CSF of amyloid-β 42 peptide (Aβ42), Aβ40, total tau, and phosphorylated tau (p-tau) were realized using the standardized commercially available INNOTEST sandwich enzyme-linked immunosorbent assay (Fujirebio, Ghent, Belgium).

### Cognitive testing

At baseline, participants were administered a neuropsychological test battery that included the four following cognitive tests: (1) the Free and Cued Selective Reminding Test (FCSRT) [39], measuring verbal episodic memory. In this associative memory test, an individual has to learn 16 words by groups of four with each corresponding cue provided verbally by the tester (e.g. “fish” is the cue for the word “herring”). Here, we used the sum of the three free recalls: (2) the Verbal Fluency (VF) test [40], which consists in producing as many words (animals) as possible within 2 min, assessing lexical access and semantic memory; (3) the Trail Making Test B (TMT-B) [41], measuring attention and executive functioning by recording the time in seconds to complete the task; and (4) the Rey–Osterrieth Complex Figure test [42], assessing visuospatial and visuoconstructive abilities by reproducing complex drawing first by copying and then from memory assessed at 3 min. For the FCSRT, verbal fluency, and the Rey figure test, a higher score indicates better performance, whereas for the TMT-B test, a higher score (in seconds) indicates worse performance.

### Statistical analysis

Participants’ characteristics at baseline were described and compared according to sex using analysis of variance (means) and *χ*^2^ tests to assess differences in means and proportions, respectively. A description of ADRD biomarker distribution across ages was also performed.

#### Social and lifestyle latent factors

The first step of our modelling approach consisted in the creation of two latent global indicators constituted of early to midlife social and latelife lifestyle factors. We used Item Response Theory (IRT) models, and more specifically the Graded Response Model (GRM), a class of latent variable models that links ordered polytomous manifest variables (i.e. response) to their underlying single latent trait of interest. Each individual’s response to an item of the trait is considered as a manifestation of this trait. The latent trait value of each participant can be thought as its “ability” at the time of data collection. Ability scores for each participant can be predicted from the GRM parameters and the participant’s responses. In this work, two different measures from two separate IRT models were established based on an a priori life course hypothesis. The first targeted *early to midlife social factors*, including education level, occupation complexity, and salary, while the second targeted *latelife lifestyle factors*, comprising physical activity, leisure activities, and social network at study entry. GRM fits were assessed using two-way margins and were deemed satisfactory. We then extracted and standardized predicted scores that ranged from − 2.33 to 1.87 and − 1.71 to 2.14 for early to midlife social and latelife lifestyle indicators, respectively, with higher values representing more stimulating levels of social and lifestyle factors. Additional information regarding the two latent indicators is reported in Additional file 2.

To assess further the robustness of the two latent indicator-related results, we performed the following two sensitivity analyses: first, as correlations between social network and other components were low, we repeated our original analysis excluding social network from the latelife lifestyle latent indicator. Second, we re-ran our main model after excluding the two physical items of the leisure activity questionnaire from the latelife lifestyle indicator.

#### Mediation analysis

A pathway analysis was conducted to explore relationships between the social and lifestyle indicators, ADRD biomarkers, and cognitive functions using structural equation models (SEM). The two latent indicators established during the first step were used as exposures of interest. We hypothesized three different pathways of actions of social and lifestyle indicators on cognition: SVD, AD-specific pathology, and neurodegeneration.

- SVD was represented by a latent variable constituted of WMH volumes (standardized on total intracranial volume (TIV) and log-transformed) and Fazekas scales of paraventricular and deep WMH (see Additional file 7: Fig. S1 for latent variable details).
- AD pathology was represented by a latent variable including CSF Ab42/Ab40 ratio, CSF p-tau (log transformed), and mean global SUVr amyloid-PET (log transformed and standardized by radioligand).
- Neurodegeneration was represented by a latent variable, comprising hippocampal volume (standardized on TIV and log-transformed), cortical thickness, brain parenchymal fraction, and SUVr FDG-PET. Latent variable indicators were reverse coded so that a higher score represented greater neurodegeneration.

The outcome of interest, cognitive functioning, was represented by a latent variable constituted by the following four cognitive tests: FCSRT, Animal Fluency, Rey figure test, and TMT-B (the latter was log-transformed and inversed so that higher scores correspond to higher performances). Higher values of the latent variable represent higher cognitive performances.

**Fig. 1:**
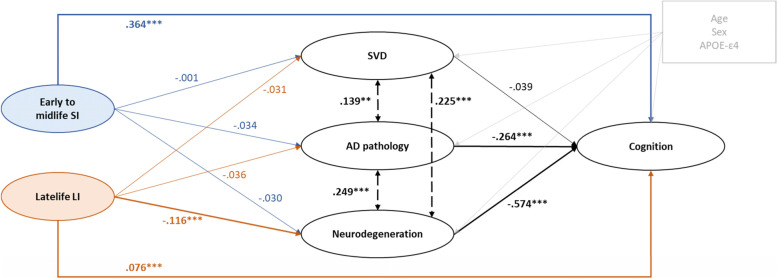
Structural equation model representing the pathways involved in the association between social and lifestyle indicators and cognition. SI, social indicator; LI, lifestyle indicator; SVD, small vessel disease; AD, Alzheimer’s disease; CI, confidence interval. Each pathway was adjusted for age, sex, and APOE status, yet they were not represented here for clarity. Solid arrows represent associations. Dashed bidirectional arrows represent correlations. Bolded arrows represent significant associations. Estimates presented in the graph are standardized. *P* values: * < 0.05, ** < 0.01, *** < 0.001. Social and lifestyle latent indicators were obtained through a graded response model, and circles represent latent variables from SEM. Variables composition: early to midlife SI: education, occupational complexity, and salary; latelife LI: physical activity, leisure activities, and social network; SVD: white matter hyperintensity volume, paraventricular white matter lesions, and deep white matter lesions; AD pathology: CSF Aβ42/Aβ40 ratio, CSF phosphorylated Tau, and SUVr amyloid-PET; neurodegeneration: hippocampal volume, cortical thickness, SUVr FDG-PET, and brain parenchymal fraction; cognition: Verbal Fluency, Free and Cued Selective Reminding test, Trail making test B, and Rey figure test

We built a SEM to test whether the association between latent life course social and lifestyle indicators and cognitive performances was mediated by AD pathology, SVD, and neurodegeneration biomarkers (Fig. 1). All potential paths were adjusted for potential confounders: age at baseline, sex, and APOE-e4 status. We used maximum likelihood estimators and full information maximum likelihood (FIML) to handle missing data under the missing at random hypothesis. Coefficients for direct and indirect effects are presented and standard errors for indirect effect parameters were computed using the Delta method. Standardized coefficients as well as 95% confidence intervals (CI) for endogenous variables (i.e. SVD, AD pathology, neurodegeneration, and cognition) and latent social and lifestyle indicators are reported. Only coefficients on associations going to a particular outcome are comparable.

Analyses were conducted in R (version 3.6.0), using ltm (version 1.1–1) [43] and lavaan (version 0.6–4) [44] packages.

## Results

A description of the sample characteristics is presented in Table 1. Participants’ mean age at baseline was 70.9 years old and 61.9% were women. Twenty-eight percent were APOE-ɛ4 carriers and 59.2% had a CDR score of 0.5. Women had on average lower educational level, occupational complexity, income, and social network index score than men. As expected, markers of SVD, AD pathology, neurodegeneration, and cognition worsened with age (Additional file 3: Table S1).

**Table 1:** Baseline characteristics of study participants by sex, the Memento study

*N* (%)	Total	Men	Women	*P* value
	*N* = 2323	*N* = 885	*N* = 1438	
Age at baseline, mean (SD)	70.9 (8.7)	71.3 (8.4)	70.6 (8.9)	0.07
APOE-e4 status				0.30
No	1538 (66.2)	571 (64.5)	967 (67.2)	
1 allele or more	658 (28.3)	267 (30.2)	391 (27.2)	
CDR				0.07
0	933 (40.2)	330 (37.3)	603 (41.9)	
0.5	1375 (59.2)	550 (62.2)	825 (57.4)	
Education level				< .001
No diploma or primary school	371 (16.0)	111 (12.5)	260 (18.1)	
Short secondary school	677 (29.1)	245 (27.7)	432 (30.0)	
Long secondary school	362 (15.6)	126 (14.2)	236 (16.4)	
Some college and higher	908 (39.1)	403 (45.6)	505 (35.1)	
Occupations				< .001
Lower	469 (20.2)	175 (19.8)	294 (20.4)	
Intermediate	1093 (47.1)	277 (31.3)	816 (56.8)	
Higher	735 (31.6)	427 (48.3)	308 (21.4)	
Salary (euros)				< .001
400–800	46 (2.0)	9 (1.0)	37 (2.6)	
800–1200	126 (5.4)	20 (2.3)	106 (7.4)	
1200–1800	311 (13.4)	75 (8.5)	236 (16.4)	
1800–2500	407 (17.5)	144 (16.3)	263 (18.3)	
2500–4000	640 (27.6)	259 (29.3)	381 (26.5)	
4000–6000	341 (14.7)	171 (19.3)	170 (11.8)	
6000 +	135 (5.8)	88 (9.9)	47 (3.3)	
Physical activity				0.65
Low	350 (15.1)	125 (14.1)	225 (15.7)	
Moderate	902 (38.8)	339 (38.3)	563 (39.2)	
Vigorous	818 (35.2)	320 (36.2)	498 (34.6)	
Leisure activity				0.19
< 6	594 (25.6)	240 (27.1)	354 (24.6)	
6–7	782 (33 .7)	311(35.1)	471 (32.7)	
8–9	580 (25.0)	208 (23.5)	372 (25.9)	
> 9	238 (10.2)	79 (8.9)	159 (11.1)	
Social network				< .001
0	174 (7.5)	38 (4.3)	136 (9.5)	
1	642 (27.6)	155 (17.5)	487 (33.9)	
2	1085 (46.7)	484 (54.7)	601 (41.8)	
3	280 (12.1)	144 (16.3)	136 (9.5)	

Missing values: APOE status = 127; CDR = 15; education = 5; occupation = 26; salary = 317; physical activity = 253; leisure activity = 129; social network = 142. Physical activity is based on the International Physical Activity Questionnaire (IPAQ) and categorized in 3 classes. Leisure activity is the number of activities realized at least once a week from a list of 15 activities, then coded in 4 classes. Social network is an index combining the following information: married (0/1), > 2 close relatives/friends (0/1), and membership in community organizations (0/1)

### Structural equation model

The final SEM model fit was acceptable with a root mean square error of approximation (RMSEA) of 0.049 (95% *CI* = 0.046–0.052), a standardized root mean square residual (SRMR) of 0.047, and a Comparative Fit Index (CFI) and Tucker-Lewis Index (TLI) of 0.942 and 0.922, respectively. Figure 1 shows the full SEM with direct estimates. Factor loadings between indicators and latent common variables, as well as explained variances are presented in Additional file 4: Table S2. Residual correlations of the model remained low (Additional file 8: Fig. S2).

Estimates of mediation effects between social and lifestyle indicators and ADRD markers are presented in Table 2. A higher level of early to midlife latent social indicator was only directly associated with better cognitive function (*β*_direct_ = 0.364 (0.322; 0.405)). Indeed, the early to midlife social indicator was not associated with SVD and AD pathology nor neurodegeneration markers. A higher level of latelife latent lifestyle indicator was associated with better cognitive function, both directly (*β*_direct_ = 0.076 (0.033; 0.118)) and indirectly (*β*_indirect_ = 0.077 (0.046; 0.108)). This association was mediated by a direct effect of the latelife lifestyle indicator on lower neurodegeneration (*β*_direct_ =  − 0.116 (− 0.153; − 0.079); *β*_indirect_ = 0.066 (0.042; − 0.090)). However, the latelife lifestyle indicator was not associated with AD pathology nor with SVD at the significance level. Total association between latent indicators and cognition was more than twice stronger for the early to midlife social indicator than for the latelife lifestyle indicator (*β*_total_ equal to respectively 0.390 (0.351; 0.429) vs 0.153 (0.112; 0.193)). Higher AD pathology and neurodegeneration, but not SVD, were directly associated with lower cognitive function (*β*_direct_AD_ =  − 0.264 (− 0.362; − 0.165) and *β*_direct_N_ =  − 0.574 (− 0.665; − 0.483)). AD pathology, SVD, and neurodegeneration were all three positively correlated (Table 2). Sensitivity analysis excluding social network from the latelife lifestyle indicator yielded similar results compared to the primary analysis (Additional file 5: Table S3). In addition, analysis excluding physical leisure activity items showed results similar to the main analysis, with slightly higher effect sizes for the direct effect between the latelife lifestyle indicator and SVD and AD pathology, but not significant (Additional file 4: Table S4).

**Table 2:** Estimates of the direct and indirect effects of social and lifestyle indicators on ADRD biomarkers and cognitive performance using structural equation models

**From**	To	*β*	(95% CI)	*P* value
***Direct effects***				
Early to midlife SI	SVD	− 0.001	(− 0.042; 0.041)	0.968
	AD pathology	− 0.034	(− 0.104; 0.036)	0.339
	Neurodegeneration	− 0.030	(− 0.068; 0.008)	0.121
	Cognition	0.364	(0.322; 0.405)	0.000
Latelife LI	SVD	− 0.031	(− 0.072; 0.009)	0.129
	AD pathology	− 0.036	(− 0.105; 0.032)	0.297
	Neurodegeneration	− 0.116	(− 0.153; − 0.079)	0.000
	Cognition	0.076	(0.033; 0.118)	0.000
AD pathology	Cognition	− 0.264	(− 0.362; − 0.165)	0.000
SVD	Cognition	− 0.039	(− 0.093; 0.014)	0.149
Neurodegeneration	Cognition	− 0.574	(− 0.665; − 0.483)	0.000
***Indirect effects***				
Early to midlife SI	Cognition through AD pathology	0.009	(− 0.010; 0.028)	0.344
	Cognition through SVD	0.000	(− 0.002; 0.002)	0.968
	Cognition through neurodegeneration	0.017	(− 0.005; 0.039)	0.124
Latelife LI	Cognition through AD pathology	0.010	(− 0.009; 0.028)	0.308
	Cognition through SVD	0.001	(− 0.001; 0.004)	0.296
	Cognition through neurodegeneration	0.066	(0.042; 0.090)	0.000
***Correlations***				
AD pathology	Neurodegeneration	0.249	(0.143; 0.355)	0.000
AD pathology	SVD	0.139	(0.052; 0.227)	0.002
SVD	Neurodegeneration	0.225	(0.167; 0.283)	0.000

Latent variables composition: early to midlife SI: education, occupational complexity, and salary; latelife LI: physical activity, leisure activities, and social network; SVD: white matter hyperintensity volume, paraventricular white matter lesions, and deep white matter lesions; AD pathology: CSF Aβ42/Aβ40 ratio, CSF phosphorylated Tau, and SUVr amyloid-PET; neurodegeneration: hippocampal volume, cortical thickness, SUVr FDG-PET, and brain parenchymal fraction; cognition: Verbal Fluency, Free and Cued Selective Reminding test, Trail making test B, and Rey figure test

*SI* social indicator, *LI* lifestyle indicator, *SVD* small vessel disease, *AD* Alzheimer’s disease, *CI* confidence interval

Associations between covariates and ADRD markers are presented in Table 3. Women had lower neurodegeneration (*β*_direct_ =  − 0.603 (− 0.677; − 0.529)) and higher cognitive function (*β*_total_ = 0.186 (0.100; 0.273)). Increasing age was directly associated with worse SVD, AD pathology, and neurodegeneration. It was associated with worse cognition in total (*β*_total_ =  − 0.042 (− 0.047; − 0.038)). APOE-e4 carrier status was directly associated with higher AD pathology (*β*_direct_ = 0.835 (0.703; 0.967)) and slightly with SVD (*β*_direct_ = 0.085 (− 0.004; 0.174)). It was indirectly associated with lower cognitive function through worse AD pathology (*β*_indirect_ =  − 0.338 (− 0.438; − 0.237)).

**Table 3:** Estimates of the direct and indirect effects of covariates on ADRD biomarkers and cognitive performances using structural equation models

**From**	To	***β***	(95% CI)	*P* value
***Direct effects***				
Women	SVD	0.016	(− 0.068; 0.100)	0.715
	AD pathology	− 0.129	(− 0.266; 0.008)	0.067
	Neurodegeneration	− 0.603	(− 0.677; − 0.529)	0.000
	Cognition	− 0.193	(− 0.296; − 0.091)	0.000
Age	SVD	0.053	(0.049; 0.057)	0.000
	AD pathology	0.048	(0.041; 0.055)	0.000
	Neurodegeneration	0.075	(0.072; 0.078)	0.000
	Cognition	0.015	(0.007; 0.024)	0.001
APOE e4 carrier	SVD	0.085	(− 0.004; 0.009)	0.063
	AD pathology	0.835	(0.703; 0.967)	0.000
	Neurodegeneration	0.199	(0.117; 0.280)	0.000
	Cognition	0.023	(− 0.097; 0.143)	0.709
***Indirect effects***				
Women	Cognition through AD pathology	0.034	(− 0.004; 0.07)	0.085
	Cognition through SVD	− 0.001	( − 0.004; 0.003)	0.724
	Cognition through neurodegeneration	0.346	(0.274; 0.418)	0.000
Age	Cognition through AD pathology	− 0.013	( − 0.018; − 0.007)	0.000
	Cognition through SVD	− 0.002	(− 0.005; 0.001)	0.150
	Cognition through neurodegeneration	− 0.043	(− 0.050; − 0.036)	0.000
APOE e4 carrier	Cognition through AD pathology	− 0.220	(− 0.312; − 0.129)	0.000
	Cognition through SVD	− 0.003	(− 0.009; 0.002)	0.255
	Cognition through neurodegeneration	− 0.114	(− 0.165; − 0.064)	0.000

Latent variable composition: early to midlife CR: education, occupational complexity, and salary; latelife CR: physical activity, leisure activities, and social network; SVD: white matter hyperintensity volume, paraventricular white matter lesions, and deep white matter lesions; AD pathology: CSF Aβ42/Aβ40 ratio, CSF phosphorylated Tau, and SUVr amyloid-PET; neurodegeneration: hippocampal volume, cortical thickness, SUVr FDG-PET, and brain parenchymal fraction; cognition: Verbal Fluency, Free and Cued Selective Reminding test, Trail making test B, and Rey figure test

*RI* resilience indicator, *SVD* small vessel disease, *AD* Alzheimer’s disease, *CI* confidence interval

## Discussion

In this cross-sectional analysis of data from a clinic-based study, we aimed at understanding the role of different ADRD biomarkers in the association between factors known to enhance resilience over the life course and cognitive function at older ages. Our results suggest that social and lifestyle factors favour cognitive performances, directly for both early and latelife factors, and also indirectly for latelife lifestyle factors, through lower neurodegeneration. Moreover, our work highlights the importance of investigating different factors over the life course.

More stimulating social and lifestyle factors have often been associated with improved cognitive performances and lower dementia risk [8]. However, it remains unclear how higher levels of such factors may lead to improved cognitive performances at older ages. Studies identifying underlying mechanisms through multiple markers of brain pathology are thus required to better understand the influence of these factors on cognitive and brain ageing, which could ultimately inform on innovative strategies for dementia prevention. In this work, we hypothesized that social (earlylife) and lifestyle (latelife) factors may improve cognitive function either directly through potential compensation strategies or indirectly by lowering brain pathology (through cerebrovascular lesion, AD pathology, or neurodegeneration).

First, regarding the potential direct effect of social and lifestyle factors on cognitive function, it has previously been evidenced that factors such as education level, physical activities, or cognitively enhancing activities are involved in resilience against ADRD, i.e. the ability of the brain to cope against adversity and maintain “normal” cognitive functions. For instance, these factors are hypothesized to influence cognitive reserve, which allows individuals to maintain cognitive functions in the presence of brain alterations [45]. In this work, both early to midlife latent social indicator and latelife lifestyle indicator were directly associated with higher cognitive performances, independently of AD or SVD pathologies, and neurodegeneration. Yet, the effect size for the association of the latelife lifestyle indicator with cognitive functions was 4.8 times lower than for the early to midlife social indicator. These results are in agreement with the hypothesis that intellectual stimulations throughout life (mostly through education, occupation, and SES) help in maintaining cognitive performances despite brain pathology, by enhancing compensation strategies and brain network efficiency, capacity, or flexibility [46].

Then, regarding the indirect impact of social and lifestyle factors on cognition through brain pathology, we did not observe a significant association between SVD and more stimulating latelife lifestyle, contrary to previous reports in favour of a protective effect of lifestyle factors on cerebrovascular pathology [9, 14, 47, 48]. Moreover, although some studies found an association between markers of cognitive reserve and AD pathology [10–12, 14, 16, 49, 50], our results are in agreement with evidence showing no associations between social and lifestyle factors measured at different times over the lifecourse and AD pathology [9, 17, 18, 20, 25]. Our results did not evidence any mediated pathways between the early to midlife social indicator and cognitive function. On the other side, the latelife lifestyle indicator showed an indirect effect on cognition, mostly through lower neurodegeneration, in line with previous reports linking physical activity or leisure activities with hippocampal volume or other brain volume markers [47, 51–53].

Overall, our results suggest that social and lifestyle factors may influence cognition through different mechanisms. Our findings do not support a contribution of social and lifestyle factors to resistance against AD and cerebrovascular pathology given the absence of associations between the latent indicators and AD pathology or SVD markers. The divergent results across studies could be explained by variations in pathology levels and participants’ clinical status, where participants with more advanced pathology may be exhibiting lower variability, potentially explaining the lack of associations in studies with MCI patients. Moreover, variability in definitions or timing of social and lifestyle factors makes comparisons across studies difficult. Latent factors allow capturing the comprehensive effect of multiple resilience-enhancing factors over the lifespan. Our a priori hypothesis, which appears to be a strength and novelty of this work, was that differentiating the time window of exposure to social and lifestyle factors that can influence resilience may be of importance when assessing their association with cognitive decline or dementia risk [8]. In addition, if early to midlife factors are most of the time not influenced by the latelife outcome, associations between latelife factors, such as physical, cognitive, or social activities, and latelife outcomes may be the results of reverse causality.

### Limitations

This work has some limitations. First, given data are cross-sectional, causal inference must be discussed with caution, as reverse causality cannot be excluded and temporal ordering between ADRD biomarkers cannot be determined. In addition, our study sample consists of persons presenting with cognitive complaints or MCI in memory clinics. Results thus apply to a population of individuals at risk for ADRD and may not be generalizable to the general population. However, comprehensive neuroimaging and CSF markers being less widely available in population-based cohorts, our study provides valuable insights regarding the different mechanisms involved in cognitive ageing. Moreover, social and lifestyle factors were self-reported at baseline; thus, measurement bias cannot be excluded, especially for latelife lifestyle factors. Other factors (such as midlife lifestyle factors, childhood cognitive enrichment, or diet) may contribute to resilience over the life course but were not recorded. Finally, it could be argued that amyloid-PET and lumbar punctures were realized in smaller and potentially selected samples. However, to ensure the validity of our findings, we applied a Full Information Maximum Likelihood approach handling missing data under the missing at random hypothesis. Given the large number of information considered in the analysis, the missing at random assumption is plausible.

Despite these limitations, this study has important strengths and contributes to the sparse literature on mechanisms underlying the influences of social and lifestyle factors on cognition. The Memento study provides an adequate setting to investigate the mediating role of ADRD biomarkers, due to the availability of various neuroimaging and CSF markers. In addition, this study enabled us to assess different social and lifestyle factors, in line with the hypothesis that resilience is built over multiple experiences across the lifespan. Finally, structural equation models are a powerful tool to model complex relationships with brain and cognitive health as latent constructs and robustly estimate direct and indirect effects.

## Conclusions

In conclusion, this work suggests that more stimulating levels of social and lifestyle factors may be associated with better cognitive function through different mechanisms over the life course. Our results support the hypothesis that investing in education and earlylife cognitive-enhancing activities may have an impact on cognitive health later in life. Complementary findings based on longitudinal evaluation of pathological markers and cognitive function could help to build new comprehensive strategies for dementia prevention.

## Supplementary Information

**Additional file 1:** PET measurement additional information

**Additional file 2:** Early to midlife ‘social factors’ and latelife ‘lifestyle factors’ latent measures additional information

**Additional file 3:** **Table S1.** Description of ADRD biomarkers measures by age group, the Memento study

**Additional file 4:** **Table S2.** Estimates of the latent variables’ measurement equations from the structural equation model.

**Additional file 5:** **Table S3.** Estimates of the direct and indirect effects of social and lifestyle indicators on ADRD biomarkers and cognitive performance using structural equation models, excluding social network from the lifestyle indicator.

**Additional file 6:** **Table S4.** Estimates of the direct and indirect effects of social and lifestyle indicators on ADRD biomarkers and cognitive performance using structural equation models, excluding physical leisure activities items.

**Additional file 7:** **Fig. S1.** Detailed latent variables composition.

**Additional file 8:** **Fig. S2.** Residual correlations of the structural equation model.

**Additional file 9:** Memento Cohort Study Group.

## Data Availability

Memento data access request is available via the Dementia Platform UK Data Access appliance form (https://portal.dementiasplatform.uk/Apply) or via the Memento Secretariat (sophie.lamarque@u-bordeaux.fr).
