# Supplementary material for: Explaining the association between social and lifestyle factors and cognitive functions: a pathway analysis in the Memento cohort
Source: Alzheimers Res Ther. 2022 May 18;14:68. doi: 10.1186/s13195-022-01013-8 (PMC9115948; doi:10.1186/s13195-022-01013-8)
Supplement: Supplementary file 1 — Additional file 1: PET measurement additional information [file 13195_2022_1013_MOESM1_ESM.docx]

**Additional file 1: PET measurement additional information**

FDG-PET was performed in 57% of participants. Brain FDG-PET scans were acquired 30 minutes after injection of 2 MBq/kg of 2-deoxy-2-18F-fluoro-D-glucose. All acquisitions consisted of 3×5 minute frames.

Amyloid PET examinations were performed in 28% of participants as part of the MEMENTO-Amyging ancillary study using either Florbetapir (^18^F) or Flutemetamol (^18^F) radioligands. Florbetapir scans (3x5 min) were acquired 50min after injection of 370 (± 10%) MBq. Flutemetamol scans (4x5 min) were acquired 90min after injection of 185 (± 10%) MBq, Images were then reconstructed using an iterative algorithm, and last, frames were realigned, averaged and quality-checked by the CATI team.

All PET images were then analysed with an in-house pipeline developed by the CATI, including partial volume effect correction (PVEC), on untransformed PET images, to reduce possible quantification biases related to spatial normalization or co-registration (Habert et al. 2018).

MRI 3D T1-weighted images were segmented and spatially normalized into the MNI space using the VBM8 package (http://dbm.neuro.uni-jena.de/vbm/) implemented in SPM8. Deformation fields and grey and white matter masks were generated. Structural MRI images were co-registered to PET images using SPM8 with visual inspection to detect any co-registration errors. Using inverse deformation fields and matrix transformation, composite cortical regions of interest (ROIs) and a reference region were placed in the individual native PET space. We then applied a PVEC algorithm that performs a region-based voxel-wise (RBV) correction of the entire image, using the anatomical parcellation of MRI scans and an accurate measure of the point spread function of the PET scanner. Finally, parametric PET images were created for each individual, by dividing each voxel with the mean activity extracted from the reference region.

- For glucose metabolism PET images, a set of four AD-specific bilateral ROIs was used: posterior cingulate cortex, inferior parietal lobule, precuneus and inferior temporal gyrus. These ROIs corresponded to significant clusters obtained from a voxel-based comparison performed with SPM5 ((<http://www.fil.ion.ucl.ac.uk/spm/))> between a group of 40 healthy controls and a group of 40 patients with clinical probable AD. Data used for this step were taken from the Alzheimer's Disease Neuroimaging Initiative database (adni.loni.ucla.edu). Images were then scaled in intensity using the global mean value of the average value of the pons.
- For amyloid PET images, standard uptake value ratios (SUVR) were calculated in target areas such as the medial frontal cortex, temporal cortex, parietal cortex, posterior cingulate cortex, anterior cingulate cortex and the precuneus, using a composite region of whole cerebellum and pons as a reference region (Habert et al, 2018).
