# Supplementary material for: Explaining the association between social and lifestyle factors and cognitive functions: a pathway analysis in the Memento cohort
Source: Alzheimers Res Ther. 2022 May 18;14:68. doi: 10.1186/s13195-022-01013-8 (PMC9115948; doi:10.1186/s13195-022-01013-8)
Supplement: Supplementary file 2 — Additional file 2: Early to midlife ‘social factors’ and latelife ‘lifestyle factors’ latent measures additional information [file 13195_2022_1013_MOESM2_ESM.docx]

**Additional file 2.** **Early to midlife ‘social factors’ and latelife ‘lifestyle factors’ latent measures additional information**

Two latent indicators of ‘social’ factors (i.e. education level, occupational complexity, and salary) and lifestyle factors (i.e. physical activity, leisure activities, and social network) have been created using graded response model (GRM), a specific Item response theory model for ordered polytomous data. The ‘social factors’ indicator is thought to represent early to midlife social level, while the ‘lifestyle factors’ indicator is thought to represent latelife lifestyle. Correlations between each factor of the two latent indicators are presented below. GRM provides discrimination values for each factor, which describe how well each factor can differentiate between individuals at different trait levels. For the early to midlife ‘social factors’ indicator, discriminations values are of 2.44 for education, 1.97 for occupational complexity, and 1.34 for salary. For the latelife ‘lifestyle factors’ indicator, discrimination values are of 0.48 for physical activity, 2.67 for leisure activities, and 0.45 for social network.

| Correlation coefficients (Kendall’s tau coefficient) between components of the early to midlife ‘social factors’ indicator. | | | |
| --- | --- | --- | --- |
|  | Education level | Occupational complexity | Salary |
| Education level | - | 0.52 | 0.43 |
| Occupational complexity | <.001 | - | 0.39 |
| Salary | <.001 | <.001 | - |

Upper diagonal part contains correlation coefficient estimates. Lower diagonal part contains corresponding p-values

| Correlation coefficients (Kendall’s tau coefficient) between components of the latelife ‘lifestyle factors’ indicator. | | | |
| --- | --- | --- | --- |
|  | Physical activity | Leisure activities | Social network |
| Physical activity | - | 0.18 | 0.05 |
| Leisure activities | <.001 | - | 0.19 |
| Social network | 0.02 | <.001 | - |

Upper diagonal part contains correlation coefficient estimates. Lower diagonal part contains corresponding p-values
