# Supplementary material for: Explaining the association between social and lifestyle factors and cognitive functions: a pathway analysis in the Memento cohort
Source: Alzheimers Res Ther. 2022 May 18;14:68. doi: 10.1186/s13195-022-01013-8 (PMC9115948; doi:10.1186/s13195-022-01013-8)
Supplement: Supplementary file 3 — Additional file 3: Table S1. Description of ADRD biomarkers measures by age group, the Memento study [file 13195_2022_1013_MOESM3_ESM.docx]

| Additional file Table S1: Description of ADRD biomarkers measures by age group, the Memento study | | | | | |
| --- | --- | --- | --- | --- | --- |
|  | N | Mean (sd) | | | P value |
|  |  | (- 64) | (65 – 74) | (75 +) |  |
| Small vessel disease |  |  |  |  |  |
| WMH volume | 2104 | 5.0 (8.3) | 8.7 (11.6) | 14.3 (15.7) | <0.001 |
| Paraventricular WM lesions | 2166 | 1.1 (0.4) | 1.3 (0.6) | 1.6 (0.7) | <0.001 |
| Deep WM lesions | 2166 | 0.8 (0.6) | 1.1 (0.7) | 1.5 (0.8) | <0.001 |
| AD pathology |  |  |  |  |  |
| AB 42/AB40 ratio | 402 | 0.1 (0.04) | 0.08 (0.03) | 0.07 (0.03) | <0.001 |
| Phosphorylated tau | 410 | 58.9 (33.3) | 63.7 (30.6) | 67.9 (27.0) | 0.09 |
| Global SUVr amyloid-PET | 649 | 0.7 (0.2) | 0.8 (0.3) | 0.9 (0.3) | <0.001 |
| Neurodegeneration |  |  |  |  |  |
| Hippocampal Volume | 2130 | 5.8 (0.6) | 5.5 (0.7) | 5.0 (0.8) | <0.001 |
| Cortical thickness | 2163 | 2.7 (0.1) | 2.6 (0.1) | 2.5 (0.1) | <0.001 |
| SUVr FDG-PET | 1320 | 1.8 (0.2) | 1.7 (0.2) | 1.7 (0.2) | <0.001 |
| Brain Parenchymal Fraction | 2183 | 76.4 (6.0) | 71.8 (5.9) | 67.8 (5.2) | <0.001 |
| Cognition |  |  |  |  |  |
| Verbal Fluency | 2275 | 30.2 (8.9) | 28.9 (8.8) | 26.3 (8.2) | <0.001 |
| FCSRT | 2302 | 28.7 (7.0) | 26.5 (8.0) | 23.4 (8.8) | <0.001 |
| Trail Making Test B | 2224 | 3.9 (2.1) | 4.8 (3.0) | 6.1 (4.1) | <0.001 |
| Rey Figure Test | 2155 | 17.2 (6.3) | 15.6 (7.0) | 13.2 (6.9) | <0.001 |
| WMH: White Matter Hyperintensities ; AD: Alzheimer’s Disease ; AB: amyloid-beta ; SUVr: Standardized Uptake Value ratio ; FDG-PET: fluoroDeoxyGlucose Positron Emission Tomography ; FCSRT : Free and Cued Selective Reminding test | | | | | |
