# Supplementary material for: Explaining the association between social and lifestyle factors and cognitive functions: a pathway analysis in the Memento cohort
Source: Alzheimers Res Ther. 2022 May 18;14:68. doi: 10.1186/s13195-022-01013-8 (PMC9115948; doi:10.1186/s13195-022-01013-8)
Supplement: Supplementary file 4 — Additional file 4: Table S2. Estimates of the latent variables’ measurement equations from the structural equation model. [file 13195_2022_1013_MOESM4_ESM.docx]

| Additional file Table S2: Estimates of the latent variables’ measurement equations from the structural equation model. | | |
| --- | --- | --- |
| Variables | **Factor loading** | **Measurement error variance** |
|  | **λ (95% CI)** | **δ^2^ (95% CI)** |
| Small vessel disease |  |  |
| WMH volume | 0.869 (0.854 ; 0.884) | 0.755 (0.729 ; 0.781) |
| Paraventricular WM lesions | 0.839 (0.823 ; 0.855) | 0.704 (0.677 ; 0.731) |
| Deep WM lesions | 0.881 (0.867 ; 0.900) | 0.776 (0.752 ; 0.810) |
| AD pathology |  |  |
| AB42/AB40 ratio | 0.876 (0.825 ; 0.927) | 0.767 (0.680 ; 0.859) |
| Phosphorylated tau | 0.723 (0.665 ; 0.780) | 0.523 (0.442 ; 0.608) |
| Global SUVr amyloid-PET | 0.782 (0.718 ; 0.846) | 0.612 (0.516 ; 0.716) |
| Neurodegeneration |  |  |
| Hippocampal Volume | 0.715 (0.689 ; 0.742) | 0.511 (0.474 ; 0.551) |
| Cortical thickness | 0.534 (0.500 ; 0.569) | 0.288 (0.250 ; 0.324) |
| SUVr FDG-PET | 0.613 (0.574 ; 0.652) | 0.376 (0.329 ; 0.425) |
| Brain Parenchymal Fraction | 0.757 (0.733 ; 0.781) | 0.573 (0.537 ; 0.610) |
| Cognition |  |  |
| Verbal Fluency | 0.634 (0.603 ; 0.665) | 0.402 (0.364 ; 0.442) |
| FCSRT | 0.702 (0.672 ; 0.731) | 0.493 (0.452 ; 0.534) |
| Trail Making Test B | 0.667 (0.635 ; 0.697) | 0.445 (0.403 ; 0.486) |
| Rey Figure Test | 0.600 (0.565 ; 0.634) | 0.360 (0.319 ; 0.402) |
| WMH: White Matter Hyperintensities ; AD: Alzheimer’s Disease ; AB: amyloid-beta ; SUVr: Standardized Uptake Value ratio ; FDG-PET: fluoroDeoxyGlucose Positron Emission Tomography ; FCSRT : Free and Cued Selective Reminding test | | |
