# Supplementary material for: Explaining the association between social and lifestyle factors and cognitive functions: a pathway analysis in the Memento cohort
Source: Alzheimers Res Ther. 2022 May 18;14:68. doi: 10.1186/s13195-022-01013-8 (PMC9115948; doi:10.1186/s13195-022-01013-8)
Supplement: Supplementary file 5 — Additional file 5: Table S3. Estimates of the direct and indirect effects of social and lifestyle indicators on ADRD biomarkers and cognitive performance using structural equation models, excluding social network from the lifestyle indicator. [file 13195_2022_1013_MOESM5_ESM.docx]

| Additional file 5 Table S3: Estimates of the direct and indirect effects of social and lifestyle indicators on ADRD biomarkers and cognitive performance using structural equation models, excluding social network from the lifestyle indicator. | | | | |
| --- | --- | --- | --- | --- |
| From | **To** | **β** | **(95% CI)** | **P value** |
| *Direct effects* | |  |  |  |
| Early to midlife SI | SVD | -0.002 | (-0.043 ; 0.040) | 0.937 |
|  | AD pathology | -0.035 | (-0.105 ; 0.035) | 0.331 |
|  | Neurodegeneration | -0.032 | (-0.070 ; 0.006) | 0.100 |
|  | Cognition | 0.365 | (0.324 ; 0.406) | 0.000 |
| Latelife LI | SVD | -0.029 | (-0.069 ; 0.011) | 0.161 |
|  | AD pathology | -0.037 | (-0.105 ; 0.031) | 0.291 |
|  | Neurodegeneration | -0.114 | (-0.151 ; -0.077) | 0.000 |
|  | Cognition | 0.071 | (0.028 ; 0.113) | 0.001 |
| AD pathology | Cognition | -0.264 | (-0.362 ; -0.165) | 0.000 |
| SVD | Cognition | -0.039 | (-0.093 ; 0.014) | 0.148 |
| Neurodegeneration | Cognition | -0.576 | (-0.666 ; -0.485) | 0.000 |
| *Indirect effects* |  |  |  |  |
| Early to midlife SI | Cognition through AD pathology | 0.009 | (-0.009 ; 0.028) | 0.337 |
|  | Cognition through SVD | 0.000 | (-0.002 ; 0.002) | 0.937 |
|  | Cognition through Neurodegeneration | 0.018 | (-0.004 ; 0.040) | 0.104 |
| Latelife LI | Cognition through AD pathology | 0.010 | (-0.009 ; 0.028) | 0.302 |
|  | Cognition through SVD | 0.001 | (-0.001 ; 0.003) | 0.314 |
|  | Cognition through Neurodegeneration | 0.065 | (0.042 ; 0.089) | 0.000 |
| *Correlations* |  |  |  |  |
| AD pathology | Neurodegeneration | 0.249 | (0.143 ; 0.355) | 0.000 |
| AD pathology | SVD | 0.139 | (0.052 ; 0.227) | 0.002 |
| SVD | Neurodegeneration | 0.225 | (0.167 ; 0.283) | 0.000 |
| SI: Social indicator ; LI: Lifestyle indicator ; SVD: Small Vessel Disease ; AD: Alzheimer’s Disease ; CI: Confidence Interval  Latent variables composition: Early to midlife SI: education, occupational complexity, and salary; Latelife LI: physical activity, and leisure activities; SVD: White matter hyperintensities volume, paraventricular white matter lesions, and deep white matter lesions; AD pathology: CSF Aβ42/Aβ40 ratio, CSF phosphorylated Tau, and SUVr amyloid-PET; Neurodegeneration: Hippocampal volume, Cortical thickness, SUVr FDG-PET, and Brain parenchymal fraction; Cognition: Verbal fluency, Free and Cued Selective Reminding test , Trail making test B, and Rey figure test. | | | | |
