# Supplementary material for: Explaining the association between social and lifestyle factors and cognitive functions: a pathway analysis in the Memento cohort
Source: Alzheimers Res Ther. 2022 May 18;14:68. doi: 10.1186/s13195-022-01013-8 (PMC9115948; doi:10.1186/s13195-022-01013-8)
Supplement: Supplementary file 6 — Additional file 6: Table S4. Estimates of the direct and indirect effects of social and lifestyle indicators on ADRD biomarkers and cognitive performance using structural equation models, excluding physical leisure activities items. [file 13195_2022_1013_MOESM6_ESM.docx]

| Additional file 6 Table S4: Estimates of the direct and indirect effects of social and lifestyle indicators on ADRD biomarkers and cognitive performance using structural equation models, excluding physical leisure activities items. | | | | |
| --- | --- | --- | --- | --- |
| From | **To** | **β** | **(95% CI)** | **P value** |
| *Direct effects* | |  |  |  |
| Early to midlife SI | SVD | 0.000 | (-0.041 ; 0.042) | 0.988 |
|  | AD pathology | -0.031 | (-0.101 ; 0.039) | 0.382 |
|  | Neurodegeneration | -0.030 | (-0.069 ; 0.008) | 0.117 |
|  | Cognition | 0.362 | (0.321 ; 0.404) | 0.000 |
| Latelife LI | SVD | -0.038 | (-0.079 ; 0.002) | 0.065 |
|  | AD pathology | -0.058 | (-0.125 ; 0.010) | 0.094 |
|  | Neurodegeneration | -0.117 | (-0.154 ; -0.080) | 0.000 |
|  | Cognition | 0.082 | (0.040 ; 0.125) | 0.000 |
| AD pathology | Cognition | -0.261 | (-0.360 ; -0.163) | 0.000 |
| SVD | Cognition | -0.039 | (-0.092 ; 0.014) | 0.151 |
| Neurodegeneration | Cognition | -0.572 | (-0.663 ; -0.482) | 0.000 |
| *Indirect effects* |  |  |  |  |
| Early to midlife SI | Cognition through AD pathology | 0.008 | (-0.010 ; 0.027) | 0.386 |
|  | Cognition through SVD | 0.000 | (-0.002 ; 0.002) | 0.988 |
|  | Cognition through Neurodegeneration | 0.017 | (-0.004 ; 0.039) | 0.121 |
| Latelife LI | Cognition through AD pathology | 0.015 | (-0.004 ; 0.034) | 0.116 |
|  | Cognition through SVD | 0.001 | (-0.001 ; 0.004) | 0.257 |
|  | Cognition through Neurodegeneration | 0.067 | (0.043 ; 0.091) | 0.000 |
| *Correlations* |  |  |  |  |
| AD pathology | Neurodegeneration | 0.247 | (0.141 ; 0.353) | 0.000 |
| AD pathology | SVD | 0.138 | (0.050 ; 0.225) | 0.002 |
| SVD | Neurodegeneration | 0.223 | (0.166 ; 0.281) | 0.000 |
| SI: Social indicator ; LI: Lifestyle indicator ; SVD: Small Vessel Disease ; AD: Alzheimer’s Disease ; CI: Confidence Interval  Latent variables composition: Early to midlife SI: education, occupational complexity, and salary; Latelife LI: physical activity, leisure activities, and social network; SVD: White matter hyperintensities volume, paraventricular white matter lesions, and deep white matter lesions; AD pathology: CSF Aβ42/Aβ40 ratio, CSF phosphorylated Tau, and SUVr amyloid-PET; Neurodegeneration: Hippocampal volume, Cortical thickness, SUVr FDG-PET, and Brain parenchymal fraction; Cognition: Verbal fluency, Free and Cued Selective Reminding test , Trail making test B, and Rey figure test. | | | | |
