# Supplementary material for: Explaining the association between social and lifestyle factors and cognitive functions: a pathway analysis in the Memento cohort
Source: Alzheimers Res Ther. 2022 May 18;14:68. doi: 10.1186/s13195-022-01013-8 (PMC9115948; doi:10.1186/s13195-022-01013-8)
Supplement: Supplementary file 7 — Additional file 7: Fig. S1. Detailed latent variables composition. [file 13195_2022_1013_MOESM7_ESM.docx]

**Additional file 7 Figure S1:** Detailed latent variables composition.


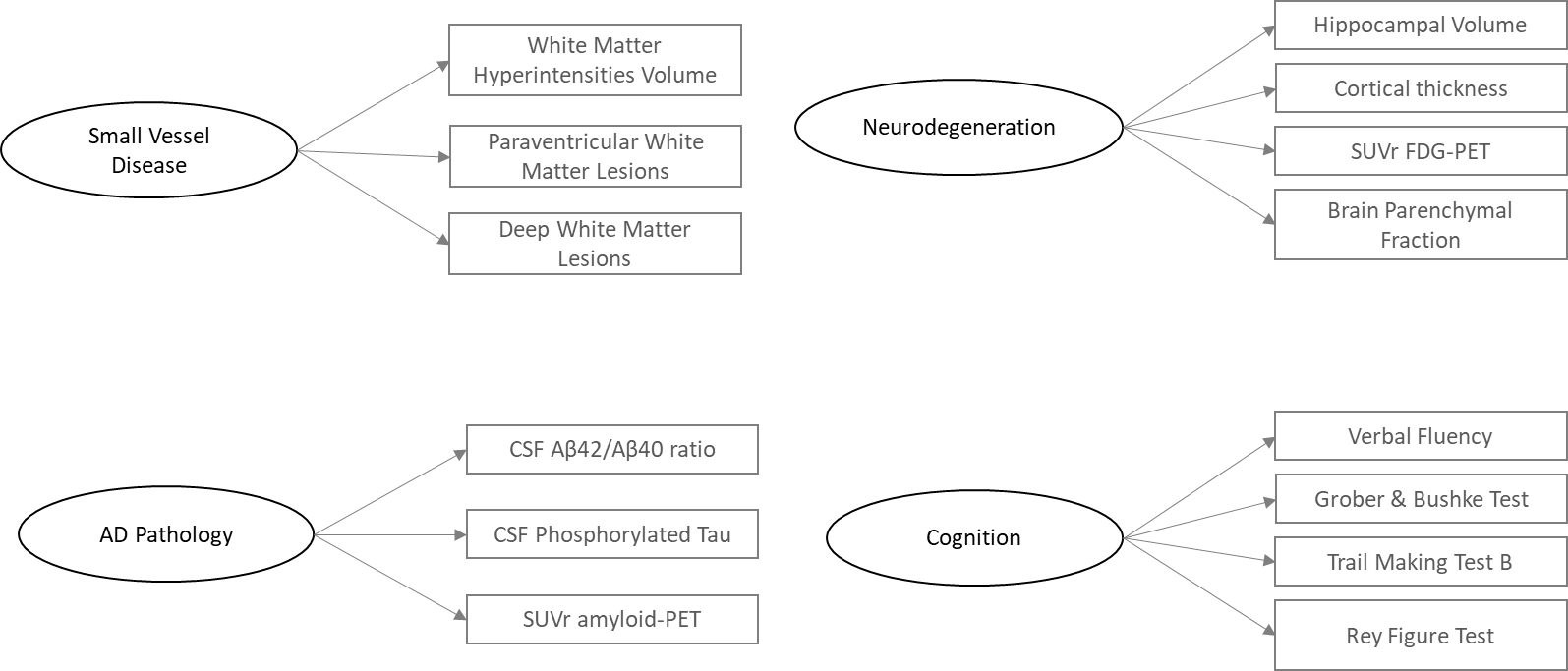


Abbreviations: WMH: White Matter Hyperintensities ; AB: amyloid-beta ; SUVr: Standardized Uptake Value ratio ; FDG-PET: fluoroDeoxyGlucose Positron Emission Tomography FCSRT: Free and Cued Selective Reminding Test
