# Supplementary material for: Explaining the association between social and lifestyle factors and cognitive functions: a pathway analysis in the Memento cohort
Source: Alzheimers Res Ther. 2022 May 18;14:68. doi: 10.1186/s13195-022-01013-8 (PMC9115948; doi:10.1186/s13195-022-01013-8)
Supplement: Supplementary file 8 — Additional file 8: Fig. S2. Residual correlations of the structural equation model. [file 13195_2022_1013_MOESM8_ESM.docx]

**Additional file 8 Figure S2:** Residual correlations of the structural equation model.


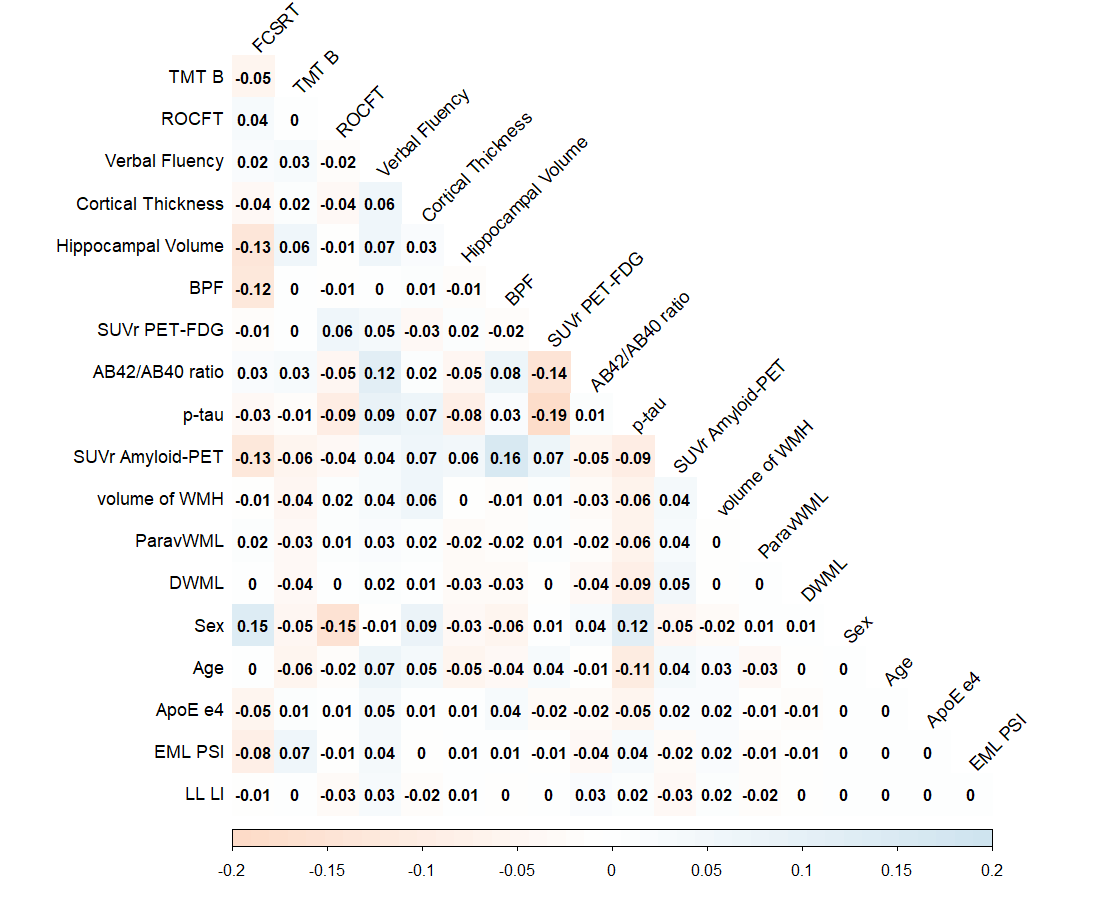


Abbreviations: WMH: White Matter Hyperintensities ; WML: White Matter Lesion ; AB: amyloid-beta ; SUVr: Standardized Uptake Value ratio ; FDG-PET: fluoroDeoxyGlucose Positron Emission Tomography ; TMT: Trail Making Test ; ROCFT: Rey-Osterrieth complex figure test ; FCSRT: Free and Cued Selective Reminding Test ; BPF: Brain Parenchymal Fraction; APOE: ApolipoProtein E ; EML: Early to MidLife ; LL: LateLife ; SI: Social indicator ; LI: Lifestyle indicator
