# Supplementary material for: Explaining the association between social and lifestyle factors and cognitive functions: a pathway analysis in the Memento cohort
Source: Alzheimers Res Ther. 2022 May 18;14:68. doi: 10.1186/s13195-022-01013-8 (PMC9115948; doi:10.1186/s13195-022-01013-8)
Supplement: Supplementary file 9 — Additional file 9: Memento Cohort Study Group. [file 13195_2022_1013_MOESM9_ESM.docx]

Memento Cohort Study Group

| Name | Degree | Location | Role |
| --- | --- | --- | --- |
| Michèle Allard | MD, PhD | Memory Resource and Research Centre of Bordeaux, CHU de Bordeaux, Hôpital Xavier Arnozan, F-33000, Bordeaux, France | Co-investigator |
| Sandrine Andrieu | MD, PhD | Memory Resource and Research Centre of Toulouse, CHU de Toulouse, Hôpital La Grave-Casselardit, F-31000, Toulouse, France | Co-investigator |
| Pierre Anthony | MD, PhD | Memory Resource and Research Centre of Colmar, Hôpitaux Civils de Colmar, F-68000, Colmar, France | Co-investigator |
| Christine Astier | MD | Memory Resource and Research Centre of Strasbourg, Hôpitaux Universitaires de Strasbourg, F-67000, Strasbourg, France | Co-investigator |
| Alexandre Augier | MD, PhD | Memory Clinic, Hôpital Avicenne, AP-HP, Hôpitaux Universitaires Paris-Seine-Saint-Denis, F-93009, Bobigny, France | Co-investigator |
| Nicolas Auguste | MD | Memory Resource and Research Centre of Saint-Etienne, CHU de Saint-Etienne, Hôpital de la Charité, F-42000, Saint-Etienne, France | Co-investigator |
| Sophie Auriacombe | MD, PhD | Memory Resource and Research Centre of Bordeaux, CHU de Bordeaux, Hôpital Pellegrin, F-33000, Bordeaux, France | Co-investigator |
| John Avet | MD, PhD | Memory Resource and Research Centre of Saint-Etienne, CHU de Saint-Etienne, Hôpital Nord, F-42000, Saint-Etienne, France | Co-investigator |
| Olivier Bailon | MD, PhD | Memory Clinic, Hôpital Avicenne, AP-HP, Hôpitaux Universitaires Paris-Seine-Saint-Denis, F-93009, Bobigny, France | Co-investigator |
| Fabrice-Guy Barral | MD | Memory Resource and Research Centre of Saint-Etienne, CHU de Saint-Etienne, Hôpital Nord, F-42000, Saint-Etienne, France | Co-investigator |
| Jean Barré | MD | Memory Resource and Research Centre of Angers, CHU d’Angers, F-49000, Angers | Co-investigator |
| Annick Barthelaix | MD, PhD | Memory Resource and Research Centre of Angers, CHU d’Angers, F-49000, Angers | Co-investigator |
| Catherine Bayle | MD | Memory Resource and Research Centre of Paris Broca, AP-HP, Paris, France | Co-investigator |
| Olivier Beauchet |  | Memory Resource and Research Centre of Angers, CHU d’Angers, F-49000, Angers | Co-investigator |
| Catherine Belin | MD, PhD | Memory Clinic, Hôpital Avicenne, AP-HP, Hôpitaux Universitaires Paris-Seine-Saint-Denis, F-93009, Bobigny, France | Co-investigator |
| Samia Belkacem | MD | Institute of Memory and Alzheimer's Disease (IM2A), Centre for NeuroImaging Research (CENIR), Brain and Spine Institute (ICM), UMR S 1127, Department of Neurology, AP-HP, Pitié-Salpêtrière University Hospital, Sorbonne Universities, Pierre et Marie Curie University, Paris, France | Co-investigator |
| Douraied Ben Salem | MD, PhD | Memory Resource and Research Centre of Brest, CHRU de Brest, F-29000, Brest, France | Co-investigator |
| Karim Bennys | MD | Memory Resource and Research Centre of Montpellier, CHU de Montpellier, Hôpital Gui de Chauliac, F-34000, Montpellier, France | Co-investigator |
| Géraldine Bera | MD | Laboratoire d'Imagerie Biomédicale, Sorbonne Universités, UPMC Univ Paris 06, Inserm U1146, CNRS UMR 7371, France NeuroSpin, I2BM, Commissariat à l'Energie Atomique, Paris, France | Co-investigator |
| Eric Berger | MD | Memory Resource and Research Centre of Besançon, CHU de Besançon, Hôpital Jean Minjoz, Hôpital Saint-Jacques, F-25000, Besançon, France | Co-investigator |
| Marc G Berger | MD, PhD | Memory Resource and Research Centre of Clermont-Ferrand, CHU de Clermont-Ferrand, F-63000, Clermont-Ferrand, France | Co-investigator |
| Emilie Bergouin | MD | Memory Resource and Research Centre of Dijon, CHU Dijon Bourgogne, Hôpital du Bocage, Hôpital de Champmaillot, F-21000, Dijon, France | Co-investigator |
| François Bertin-Hugault | MD | Memory Resource and Research Centre of Lyon, Hospices Civils de Lyon, Hôpital des Charpennes, F-69000, Lyon, France | Co-investigator |
| Guillaume Bertrand | MD | Memory Clinic, Hôpital Avicenne, AP-HP, Hôpitaux Universitaires Paris-Seine-Saint-Denis, F-93009, Bobigny, France | Co-investigator |
| François-Xavier Bertrand | MD, PhD | Memory Resource and Research Centre of Nantes, CHU de Nantes, F-44000, Nantes, France | Co-investigator |
| Catherine Beze | MD | Memory Resource and Research Centre of Center Region, CHRU de Tours, Hôpital Bretonneau, F-37000, Tours, France | Co-investigator |
| Valérie Boilet |  | Coordinating Centre, Inserm CIC-1401 Clinical Epidemiology, CHU de Bordeaux, F-33000, Bordeaux, France | Co-investigator |
| Stéphanie Bombois | MD, PhD | Memory Resource and Research Centre of Lille, CHRU de Lille, Hôpital Roger Salengro, F-59000, Lille, France | Co-investigator |
| Alain Bonafé | MD, PhD | Memory Resource and Research Centre of Montpellier, CHU de Montpellier, Montpellier, France | Co-investigator |
| Yasmina Boudali | MD | Memory Resource and Research Centre of Paris Broca, AP-HP, Paris, France | Co-investigator |
| Hatem Bouhladour | MD, PhD | Memory Resource and Research Centre of Besançon, CHU de Besançon, Hôpital Jean Minjoz, Hôpital Saint-Jacques, F-25000, Besançon, France | Co-investigator |
| Clémence Boully | MD | Memory Resource and Research Centre of Paris Broca, AP-HP, Paris, France | Co-investigator |
| Isabelle Bourdel-Marchasson | MD, PhD | Memory Resource and Research Centre of Bordeaux, CHU de Bordeaux, Hôpital Xavier Arnozan, F-33000, Bordeaux, France | Co-investigator |
| Vincent Bouteloup | PharmD | Coordinating Centre, Inserm CIC-1401 Clinical Epidemiology, CHU de Bordeaux, F-33000, Bordeaux, France | Co-investigator |
| Claire Boutet | MD | Institute of Memory and Alzheimer's Disease (IM2A), Centre for NeuroImaging Research (CENIR), Brain and Spine Institute (ICM), UMR S 1127, Department of Neurology, AP-HP, Pitié-Salpêtrière University Hospital, Sorbonne Universities, Pierre et Marie Curie University, Paris, France | Co-investigator |
| Serge Bracard | MD, PhD | Memory Resource and Research Centre of Nancy, CHU de Nancy, F-54000, Nancy, France | Co-investigator |
| Antoine Brangier | MD | Memory Resource and Research Centre of Angers, CHU d’Angers, F-49000, Angers | Co-investigator |
| Pierre-Yves Brillet | MD, PhD | Memory Clinic, Hôpital Avicenne, AP-HP, Hôpitaux Universitaires Paris-Seine-Saint-Denis, F-93009, Bobigny, France | Co-investigator |
| Laure Caillard | MD | Memory Resource and Research Centre of Paris Broca, AP-HP, Paris, France | Co-investigator |
| Fabienne Calvas | MD | Memory Resource and Research Centre of Toulouse, CHU de Toulouse, Hôpital Purpan, F-31000, Toulouse, France | Co-investigator |
| Agnès Camus | MD | Memory Resource and Research Centre of Dijon, CHU Dijon Bourgogne, Hôpital du Bocage, Hôpital de Champmaillot, F-21000, Dijon, France | Co-investigator |
| Vincent Camus | MD, PhD | Memory Resource and Research Centre of Center Region, CHRU de Tours, Hôpital Bretonneau, F-37000, Tours, France | Co-investigator |
| Sandrine Canaple | MD | Memory Resource and Research of Amiens, CHU Amiens Picardie, F-80000, Amiens, France | Co-investigator |
| Antoine Carpentier | MD, PhD | Memory Clinic, Hôpital Avicenne, AP-HP, Hôpitaux Universitaires Paris-Seine-Saint-Denis, F-93009, Bobigny, France | Co-investigator |
| Pascaline Cassagnaud | MD | Memory Resource and Research Centre of Lille, CHRU de Lille, Hôpital Roger Salengro, F-59000, Lille, France | Co-investigator |
| Françoise Cattin | MD | Memory Resource and Research Centre of Besançon, CHU de Besançon, Hôpital Jean Minjoz, Hôpital Saint-Jacques, F-25000, Besançon, France | Co-investigator |
| Ludivine Chamard | MD | Memory Resource and Research Centre of Besançon, CHU de Besançon, Hôpital Jean Minjoz, Hôpital Saint-Jacques, F-25000, Besançon, France | Co-investigator |
| Stéphane Chanalet | MD | Memory Resource and Research Centre of Nice, CHU de Nice, Hôpital Pasteur, F-06100, Nice, France | Co-investigator |
| Mathieu Chastan | MD | Memory Resource and Research Centre of Rouen, CLCC Henri Becquerel, Rouen, France | Co-investigator |
| Sophie Chauvelier | MD | Memory Resource and Research Centre of Paris Broca, AP-HP, Paris, France | Co-investigator |
| Valérie Chauvire | MD | Memory Resource and Research Centre of Angers, CHU d’Angers, F-49000, Angers | Co-investigator |
| Samia Cheriet | MD, PhD | Memory Resource and Research Centre of Toulouse, CHU de Toulouse, Hôpital Purpan, F-31000, Toulouse, France | Co-investigator |
| Anthony Clotagatide | MD | Memory Resource and Research Centre of Saint-Etienne, CHU de Saint-Etienne, Hôpital Nord, F-42000, Saint-Etienne, France | Co-investigator |
| Emmanuel Cognat | MD, PhD | Memory Resource and Research Centre of Paris Nord, AP-HP, Paris, France | Co-investigator |
| Lora Cohen | PhD | Memory Resource and Research Centre of Grenoble, CHU de Grenoble Alpes, Grenoble, France | Co-investigator |
| Jean-Marc Constans | MD, PhD | Memory Resource and Research of Amiens, CHU Amiens Picardie, F-80000, Amiens, France | Co-investigator |
| Marie-Hélène Coste | MD, PhD | Memory Resource and Research Centre of Lyon, Hospices Civils de Lyon, Hôpital des Charpennes, F-69000, Lyon, France | Co-investigator |
| Jean-Philippe Cottier | MD, PhD | Memory Resource and Research Centre of Center Region, CHRU de Tours, Hôpital Bretonneau, F-37000, Tours, France | Co-investigator |
| François Cotton | MD, PhD | Memory Resource and Research Centre of Lyon, Hospices Civils de Lyon, Hôpital des Charpennes, F-69000, Lyon, France | Co-investigator |
| Isabelle Couret | MD | Memory Resource and Research Centre of Montpellier, CHU de Montpellier, Hôpital Gui de Chauliac, F-34000, Montpellier, France | Co-investigator |
| Olivier-François Couturier | MD, PhD | Memory Resource and Research Centre of Angers, CHU d’Angers, F-49000, Angers | Co-investigator |
| Pascale Cowppli-Bony | MD, PhD | Memory Resource and Research Centre of Bordeaux, CHU de Bordeaux, Hôpital Pellegrin, F-33000, Bordeaux, France | Co-investigator |
| Véronique Cressot | MD | Memory Resource and Research Centre of Bordeaux, CHU de Bordeaux, Hôpital Xavier Arnozan, F-33000, Bordeaux, France | Co-investigator |
| Benjamin Crétin | MD | Memory Resource and Research Centre of Strasbourg, Hôpitaux Universitaires de Strasbourg, F-67000, Strasbourg, France | Co-investigator |
| Keren Danaila | MD | Memory Resource and Research Centre of Lyon, Hospices Civils de Lyon, Hôpital des Charpennes, F-69000, Lyon, France | Co-investigator |
| Jacques Darcourt | MD, PhD | Memory Resource and Research Centre of Nice, CLCC Antoine Lacassagne, Nice, France | Co-investigator |
| Jean-François Dartigues | MD, PhD | Memory Resource and Research Centre of Bordeaux, CHU de Bordeaux, Hôpital Pellegrin, F-33000, Bordeaux, France | Co-investigator |
| Ana-Maria Dascalita | MD, PhD | Memory Resource and Research Centre of Saint-Etienne, CHU de Saint-Etienne, Hôpital de la Charité, F-42000, Saint-Etienne, France | Co-investigator |
| Renaud David | MD, PhD | Memory Resource and Research Centre of Nice, CHU de Nice, Institut Claude Pompidou, F-06100, Nice, France | Co-investigator |
| Xavier De Petigny | MD | Memory Resource and Research Centre of Strasbourg, Hôpitaux Universitaires de Strasbourg, F-67000, Strasbourg, France | Co-investigator |
| Delphine De Verbizier-Lonjon | MD | Memory Resource and Research Centre of Montpellier, CHU de Montpellier, Hôpital Gui de Chauliac, F-34000, Montpellier, France | Co-investigator |
| Marielle Decousus | MD, PhD | Memory Resource and Research Centre of Saint-Etienne, CHU de Saint-Etienne, Hôpital Nord, F-42000, Saint-Etienne, France | Co-investigator |
| Isabelle Defouilloy | MD, PhD | Memory Resource and Research of Amiens, CHU Amiens Picardie, F-80000, Amiens, France | Co-investigator |
| Christine Delmaire | MD, PhD | Memory Resource and Research Centre of Lille, CHRU de Lille, Hôpital Roger Salengro, F-59000, Lille, France | Co-investigator |
| Julien Delrieu | MD | Memory Resource and Research Centre of Toulouse, CHU de Toulouse, Hôpital La Grave-Casselardit, F-31000, Toulouse, France | Co-investigator |
| Catherine Demuyinck | MD | Memory Resource and Research Centre of Strasbourg, Hôpitaux Universitaires de Strasbourg, F-67000, Strasbourg, France | Co-investigator |
| Vincent Deramecourt | MD, PhD | Memory Resource and Research Centre of Lille, CHRU de Lille, Hôpital Roger Salengro, F-59000, Lille, France | Co-investigator |
| Hervé Deramond | MD, PhD | Memory Resource and Research of Amiens, CHU Amiens Picardie, F-80000, Amiens, France | Co-investigator |
| Thomas Desmidt | MD, PhD | Memory Resource and Research Centre of Center Region, CHRU de Tours, Hôpital Bretonneau, F-37000, Tours, France | Co-investigator |
| Marie-Dominique Desruet | PharmD, PhD | Memory Resource and Research Centre of Grenoble, CHU de Grenoble Alpes, Grenoble, France | Co-investigator |
| Julien Detour |  | Memory Resource and Research Centre of Strasbourg, Hôpitaux Universitaires de Strasbourg, F-67000, Strasbourg, France | Co-investigator |
| Agnès Devendeville | MD | Memory Resource and Research of Amiens, CHU Amiens Picardie, F-80000, Amiens, France | Co-investigator |
| Mira Didic | MD, PhD | Memory Resource and Research Centre of Marseille, CHU de Marseille, Hôpital La Timone, F-13000, Marseille, France | Co-investigator |
| Maritchu Doireau | MD | Memory Resource and Research Centre of Bordeaux, CHU de Bordeaux, Hôpital Pellegrin, F-33000, Bordeaux, France | Co-investigator |
| Antonio Dos Santos | MD | Institute of Memory and Alzheimer's Disease (IM2A), Brain and Spine Institute (ICM), UMR S 1127, Department of Neurology, AP-HP, Pitié-Salpêtrière University Hospital, Sorbonne Universities, Pierre et Marie Curie University, Paris, France | Co-investigator |
| Patrice Douillet | MD | Memory Resource and Research Centre of Montpellier, CHU de Montpellier, Hôpital Gui de Chauliac, F-34000, Montpellier, France | Co-investigator |
| Foucaud Du Boisgueheneuc | MD | Memory Resource and Research Centre of Poitiers, CHU de Poitiers, Hôpital de La Milétrie, F-86000, Poitiers, France | Co-investigator |
| Delphine Dubail | MD | Memory Resource and Research Centre of Paris Broca, AP-HP, Paris, France | Co-investigator |
| Laure Ducroq-Ducastaing | MD | Memory Resource and Research Centre of Brest, CHRU de Brest, F-29000, Brest, France | Co-investigator |
| Julien Dumurgier | MD, PhD | Memory Resource and Research Centre of Paris Nord, AP-HP, Paris, France | Co-investigator |
| Diane Dupuy | MD, PhD | Memory Resource and Research of Amiens, CHU Amiens Picardie, F-80000, Amiens, France | Co-investigator |
| Emmanuelle Duron | MD, PhD | Memory Resource and Research Centre of Paris Broca, AP-HP, Paris, France | Co-investigator |
| Inna Dygai-Cochet | MD, PhD | Memory Resource and Research Centre of Dijon, CLCC Georges François Leclerc, Dijon, France | Co-investigator |
| Véronique Eder | MD, PhD | Memory Clinic, Hôpital Avicenne, AP-HP, Hôpitaux Universitaires Paris-Seine-Saint-Denis, F-93009, Bobigny, France | Co-investigator |
| Stéphane Epelbaum | MD, PhD | Institute of Memory and Alzheimer's Disease (IM2A), Brain and Spine Institute (ICM), UMR S 1127, Department of Neurology, AP-HP, Pitié-Salpêtrière University Hospital, Sorbonne Universities, Pierre et Marie Curie University, Paris, France | Co-investigator |
| Frédérique Etcharry-Bouyx | MD, PhD | Memory Resource and Research Centre of Angers, CHU d’Angers, F-49000, Angers | Co-investigator |
| Daniel Fagret | MD, PhD | Memory Resource and Research Centre of Grenoble, CHU de Grenoble Alpes, Grenoble, France | Co-investigator |
| Catherine Faisant | MD | Memory Resource and Research Centre of Toulouse, CHU de Toulouse, Hôpital La Grave-Casselardit, F-31000, Toulouse, France | Co-investigator |
| Karim Farid | MD, PhD | Memory Resource and Research Centre of Paris Nord, AP-HP, Paris, France | Co-investigator |
| Denis Fédérico | MD | Memory Resource and Research Centre of Lyon, Hospices Civils de Lyon, Hôpital des Charpennes, F-69000, Lyon, France | Co-investigator |
| Olivier Felician | MD, PhD | Memory Resource and Research Centre of Marseille, CHU de Marseille, Hôpital La Timone, F-13000, Marseille, France | Co-investigator |
| Philippe Fernandez | MD, PhD | Memory Resource and Research Centre of Bordeaux, CHU de Bordeaux, Hôpital Pellegrin, F-33000, Bordeaux, France | Co-investigator |
| Pacôme Fosse | MD | Memory Resource and Research Centre of Angers, CHU d’Angers, F-49000, Angers | Co-investigator |
| Alexandra Foubert-Samier | MD, PhD | Memory Resource and Research Centre of Bordeaux, CHU de Bordeaux, Hôpital Pellegrin, F-33000, Bordeaux, France | Co-investigator |
| Isabelle Franck | MD | Memory Resource and Research Centre of Strasbourg, Hôpitaux Universitaires de Strasbourg, F-67000, Strasbourg, France | Co-investigator |
| Monique Galitzky | MD | Memory Resource and Research Centre of Toulouse, CHU de Toulouse, Hôpital Purpan, F-31000, Toulouse, France | Co-investigator |
| Céline Gallazzini-Crepin | MD | Memory Resource and Research Centre of Grenoble, CHU de Grenoble Alpes, Grenoble, France | Co-investigator |
| Radka Gantchev | MD | Memory Resource and Research Centre of Marseille, CHU de Marseille, Hôpital La Timone, F-13000, Marseille, France | Co-investigator |
| Laurence Garbarg-Chenon | MD | Memory Clinic, Hôpital Avicenne, AP-HP, Hôpitaux Universitaires Paris-Seine-Saint-Denis, F-93009, Bobigny, France | Co-investigator |
| Guillaume Gautier | MD, PhD | Memory Resource and Research Centre of Marseille, CHU de Marseille, Hôpital La Timone, F-13000, Marseille, France | Co-investigator |
| Emmanuel Gerardin | MD, PhD | Memory Resource and Research Centre of Rouen, Neuroradiology Department, Rouen University Hospital, F-76031, Rouen, France | Co-investigator |
| Claire Gervais | MD | Memory Resource and Research Centre of Nice, CHU de Nice, Institut Claude Pompidou, F-06100, Nice, France | Co-investigator |
| Jean-Claude Getenet | MD | Memory Resource and Research Centre of Saint-Etienne, CHU de Saint-Etienne, Hôpital Nord, F-42000, Saint-Etienne, France | Co-investigator |
| Nadine Girard | MD, PhD | Memory Resource and Research Centre of Marseille, CHU de Marseille, Hôpital La Timone, F-13000, Marseille, France | Co-investigator |
| Fabienne Giraud | MD | Memory Resource and Research Centre of Marseille, CHU de Marseille, Hôpital La Timone, F-13000, Marseille, France | Co-investigator |
| Chantal Girtanner | MD | Memory Resource and Research Centre of Saint-Etienne, CHU de Saint-Etienne, Hôpital de la Charité, F-42000, Saint-Etienne, France | Co-investigator |
| Valérie Gissot | MD | Memory Resource and Research Centre of Center Region, CHRU de Tours, Hôpital Bretonneau, F-37000, Tours, France | Co-investigator |
| Caroline Grangeon | PharmD | Memory Resource and Research Centre of Nice, CHU de Nice, Institut Claude Pompidou, F-06100, Nice, France | Co-investigator |
| Daniel Grucker | MD, PhD | Memory Resource and Research Centre of Strasbourg, Hôpitaux Universitaires de Strasbourg, F-67000, Strasbourg, France | Co-investigator |
| Eric Guedj | MD, PhD | Memory Resource and Research Centre of Marseille, CHU de Marseille, Hôpital La Timone, F-13000, Marseille, France | Co-investigator |
| Claude Gueriot | MD | Memory Resource and Research Centre of Marseille, CHU de Marseille, Hôpital La Timone, F-13000, Marseille, France | Co-investigator |
| Yves Guilhermet | MD | Memory Resource and Research Centre of Lyon, Hospices Civils de Lyon, Hôpital des Charpennes, F-69000, Lyon, France | Co-investigator |
| Rémy Guillevin | MD, PhD | Memory Resource and Research Centre of Poitiers, CHU de Poitiers, Hôpital de La Milétrie, F-86000, Poitiers, France | Co-investigator |
| Sophie Haffen | MD | Memory Resource and Research Centre of Besançon, CHU de Besançon, Hôpital Jean Minjoz, Hôpital Saint-Jacques, F-25000, Besançon, France | Co-investigator |
| Didier Hannequin | MD, PhD | Memory Resource and Research Centre of Rouen, Neurology Department, Rouen University Hospital, F-76031, Rouen, France | Co-investigator |
| Sandrine Harston | MD | Memory Resource and Research Centre of Bordeaux, CHU de Bordeaux, Hôpital Xavier Arnozan, F-33000, Bordeaux, France | Co-investigator |
| Anne Hitzel | MD, PhD | Memory Resource and Research Centre of Toulouse, CHU de Toulouse, Hôpital Purpan, F-31000, Toulouse, France | Co-investigator |
| Caroline Hommet | MD, PhD | Memory Resource and Research Centre of Center Region, CHRU de Tours, Hôpital Bretonneau, F-37000, Tours, France | Co-investigator |
| Claude Hossein-Foucher | MD, PhD | Memory Resource and Research Centre of Lille, CHRU de Lille, Hôpital Roger Salengro, F-59000, Lille, France | Co-investigator |
| Fabrice Hubele | MD | Memory Resource and Research Centre of Strasbourg, Hôpitaux Universitaires de Strasbourg, F-67000, Strasbourg, France | Co-investigator |
| Agnès Jacquin-Piques | MD, PhD | Memory Resource and Research Centre of Dijon, CHU Dijon Bourgogne, Hôpital du Bocage, Hôpital de Champmaillot, F-21000, Dijon, France | Co-investigator |
| Betty Jean | MD | Memory Resource and Research Centre of Clermont-Ferrand, CHU de Clermont-Ferrand, F-63000, Clermont-Ferrand, France | Co-investigator |
| Joanne Jenn | MD, PhD | Memory Resource and Research Centre of Bordeaux, CHU de Bordeaux, Hôpital Xavier Arnozan, F-33000, Bordeaux, France | Co-investigator |
| Laure Joly | MD, PhD | Memory Resource and Research Centre of Nancy, CHU de Nancy, F-54000, Nancy, France | Co-investigator |
| Thérèse Jonveaux | MD | Memory Resource and Research Centre of Nancy, CHU de Nancy, F-54000, Nancy, France | Co-investigator |
| Adrien Julian | MD, PhD | Memory Resource and Research Centre of Poitiers, CHU de Poitiers, Hôpital de La Milétrie, F-86000, Poitiers, France | Co-investigator |
| Aurélie Kas | MD, PhD | Laboratoire d'Imagerie Biomédicale, Sorbonne Universités, UPMC Univ Paris 06, Inserm U1146, CNRS UMR 7371, France NeuroSpin, I2BM, Commissariat à l'Energie Atomique, Paris, France | Co-investigator |
| Anna Kearney-Schwartz | MD | Memory Resource and Research Centre of Nancy, CHU de Nancy, F-54000, Nancy, France | Co-investigator |
| Alice Keles | MD | Memory Resource and Research Centre of Nancy, CHU de Nancy, F-54000, Nancy, France | Co-investigator |
| Antony Kelly | MD | Memory Resource and Research Centre of Clermont-Ferrand, Centre de Lutte contre le Cancer, F-63000, Clermont-Ferrand, France | Co-investigator |
| Nathalie Keromnes | MD | Memory Resource and Research Centre of Brest, CHRU de Brest, F-29000, Brest, France | Co-investigator |
| Lejla Koric | MD | Memory Resource and Research Centre of Marseille, CHU de Marseille, Hôpital La Timone, F-13000, Marseille, France | Co-investigator |
| Alexandre Krainik | MD, PhD | Memory Resource and Research Centre of Grenoble, CHU de Grenoble Alpes, Grenoble, France | Co-investigator |
| Stéphane Kremer | MD | Memory Resource and Research Centre of Strasbourg, Hôpitaux Universitaires de Strasbourg, F-67000, Strasbourg, France | Co-investigator |
| Florian Labourée | MD | Memory Resource and Research Centre of Paris Broca, AP-HP, Paris, France | Co-investigator |
| Franck Lacoeuille | MD, PhD | Memory Resource and Research Centre of Angers, CHU d’Angers, F-49000, Angers | Co-investigator |
| Francoise Lala | MD | Memory Resource and Research Centre of Toulouse, CHU de Toulouse, Hôpital La Grave-Casselardit, F-31000, Toulouse, France | Co-investigator |
| Chantal Lamy | MD | Memory Resource and Research of Amiens, CHU Amiens Picardie, F-80000, Amiens, France | Co-investigator |
| Jean-Louis Laplanche | PharmD, PhD | Memory Resource and Research Centre of Paris Nord, AP-HP, Paris, France | Co-investigator |
| Cyrille Launay | MD, PhD | Memory Resource and Research Centre of Angers, CHU d’Angers, F-49000, Angers | Co-investigator |
| Stéphane Lehericy | MD, PhD | Institute of Memory and Alzheimer's Disease (IM2A), Centre for NeuroImaging Research (CENIR), Brain and Spine Institute (ICM), UMR S 1127, Department of Neurology, AP-HP, Pitié-Salpêtrière University Hospital, Sorbonne Universities, Pierre et Marie Curie University, Paris, France | Co-investigator |
| Sylvain Lehmann | MD, PhD | Memory Resource and Research Centre of Montpellier, CHU de Montpellier, Hôpital Gui de Chauliac, F-34000, Montpellier, France | Co-investigator |
| Hermine Lenoir | MD, PhD | Memory Resource and Research Centre of Paris Broca, AP-HP, Paris, France | Co-investigator |
| Marcel Levy | MD, PhD | Institute of Memory and Alzheimer's Disease (IM2A), Brain and Spine Institute (ICM), UMR S 1127, Department of Neurology, AP-HP, Pitié-Salpêtrière University Hospital, Sorbonne Universities, Pierre et Marie Curie University, Paris, France | Co-investigator |
| Stéphanie Libercier | MD, PhD | Memory Resource and Research Centre of Colmar, Hôpitaux Civils de Colmar, F-68000, Colmar, France | Co-investigator |
| Marie-Anne Mackowiak-Cordoliani | MD | Memory Resource and Research Centre of Lille, CHRU de Lille, Hôpital Roger Salengro, F-59000, Lille, France | Co-investigator |
| Eloi Magnin | MD | Memory Resource and Research Centre of Besançon, CHU de Besançon, Hôpital Jean Minjoz, Hôpital Saint-Jacques, F-25000, Besançon, France | Co-investigator |
| Zaza Makaroff | MD | Memory Resource and Research Centre of Lyon, Hospices Civils de Lyon, Hôpital des Charpennes, F-69000, Lyon, France | Co-investigator |
| Athina Marantidou | MD | Memory Clinic, Hôpital Avicenne, AP-HP, Hôpitaux Universitaires Paris-Seine-Saint-Denis, F-93009, Bobigny, France | Co-investigator |
| Isabelle Marcet | MD | Memory Resource and Research Centre of Bordeaux, CHU de Bordeaux, Hôpital Pellegrin, F-33000, Bordeaux, France | Co-investigator |
| Cécilia Marelli | MD, PhD | Memory Resource and Research Centre of Montpellier, CHU de Montpellier, Hôpital Gui de Chauliac, F-34000, Montpellier, France | Co-investigator |
| Sophie Marilier | MD | Memory Resource and Research Centre of Dijon, CHU Dijon Bourgogne, Hôpital du Bocage, Hôpital de Champmaillot, F-21000, Dijon, France | Co-investigator |
| Idalie Martin | MD | Memory Resource and Research Centre of Lyon, Hospices Civils de Lyon, Hôpital des Charpennes, F-69000, Lyon, France | Co-investigator |
| Olivier Martinaud | MD, PhD | Memory Resource and Research Centre of Rouen, Neurology Department, Rouen University Hospital, F-76031, Rouen, France | Co-investigator |
| Catherine Martin-Hunyadi | MD | Memory Resource and Research Centre of Strasbourg, Hôpitaux Universitaires de Strasbourg, F-67000, Strasbourg, France | Co-investigator |
| Aïcha Medjoul | MD | Memory Clinic, Hôpital Avicenne, AP-HP, Hôpitaux Universitaires Paris-Seine-Saint-Denis, F-93009, Bobigny, France | Co-investigator |
| Isabelle Merlet | MD | Memory Resource and Research Centre of Poitiers, CHU de Poitiers, Hôpital de La Milétrie, F-86000, Poitiers, France | Co-investigator |
| Danielle Mestas | MD | Memory Resource and Research Centre of Clermont-Ferrand, CHU de Clermont-Ferrand, F-63000, Clermont-Ferrand, France | Co-investigator |
| Marc-Etienne Meyer | MD, PhD | Memory Resource and Research of Amiens, CHU Amiens Picardie, F-80000, Amiens, France | Co-investigator |
| Jean-Marc Michel | MD | Memory Resource and Research Centre of Colmar, Hôpitaux Civils de Colmar, F-68000, Colmar, France | Co-investigator |
| Agnès Michon | MD | Institute of Memory and Alzheimer's Disease (IM2A), Brain and Spine Institute (ICM), UMR S 1127, Department of Neurology, AP-HP, Pitié-Salpêtrière University Hospital, Sorbonne Universities, Pierre et Marie Curie University, Paris, France | Co-investigator |
| Isabelle Migeon-Duballet | MD | Memory Resource and Research Centre of Poitiers, CHU de Poitiers, Hôpital de La Milétrie, F-86000, Poitiers, France | Co-investigator |
| Karl Mondon | MD, PhD | Memory Resource and Research Centre of Center Region, CHRU de Tours, Hôpital Bretonneau, F-37000, Tours, France | Co-investigator |
| Clément Morgat | PharmD, PhD | Memory Resource and Research Centre of Bordeaux, CHU de Bordeaux, Hôpital Pellegrin, F-33000, Bordeaux, France | Co-investigator |
| Véronique Moullart | MD | Memory Resource and Research of Amiens, CHU Amiens Picardie, F-80000, Amiens, France | Co-investigator |
| Christian Moussard | MD | Memory Resource and Research Centre of Besançon, CHU de Besançon, Hôpital Jean Minjoz, Hôpital Saint-Jacques, F-25000, Besançon, France | Co-investigator |
| Aurélie Mouton | MD, PhD | Memory Resource and Research Centre of Nice, CHU de Nice, Institut Claude Pompidou, F-06100, Nice, France | Co-investigator |
| Izzie Jacques Namer | MD, PhD | Memory Resource and Research Centre of Strasbourg, Hôpitaux Universitaires de Strasbourg, F-67000, Strasbourg, France | Co-investigator |
| Georges Niewiadomski | MD, PhD | Memory Resource and Research Centre of Nice, CHU de Nice, Institut Claude Pompidou, F-06100, Nice, France | Co-investigator |
| Guillaume Nivaggioni | MD | Memory Resource and Research Centre of Nice, CHU de Nice, Institut Claude Pompidou, F-06100, Nice, France | Co-investigator |
| Marie Noblet | MD, PhD | Memory Resource and Research Centre of Strasbourg, Hôpitaux Universitaires de Strasbourg, F-67000, Strasbourg, France | Co-investigator |
| Michel Nonent | MD, PhD | Memory Resource and Research Centre of Brest, CHRU de Brest, F-29000, Brest, France | Co-investigator |
| Fati Nourhashemi | MD, PhD | Memory Resource and Research Centre of Toulouse, CHU de Toulouse, Hôpital La Grave-Casselardit, F-31000, Toulouse, France | Co-investigator |
| Hélène Oesterle | MD | Memory Resource and Research Centre of Colmar, Hôpitaux Civils de Colmar, F-68000, Colmar, France | Co-investigator |
| Galdric Orvoen | MD | Memory Resource and Research Centre of Paris Broca, AP-HP, Paris, France | Co-investigator |
| Pierre Jean Ousset | MD, PhD | Memory Resource and Research Centre of Toulouse, CHU de Toulouse, Hôpital La Grave-Casselardit, F-31000, Toulouse, France | Co-investigator |
| Amandine Pallardy | MD | Memory Resource and Research Centre of Nantes, CHU de Nantes, F-44000, Nantes, France | Co-investigator |
| Claire Paquet | MD, PhD | Memory Resource and Research Centre of Paris Nord, AP-HP, Paris, France | Co-investigator |
| Pierre-Yves Pare | MD, PhD | Memory Resource and Research Centre of Angers, CHU d’Angers, F-49000, Angers | Co-investigator |
| Anne Pasco | MD, PhD | Memory Resource and Research Centre of Angers, CHU d’Angers, F-49000, Angers | Co-investigator |
| Pierre Payoux | MD, PhD | Memory Resource and Research Centre of Toulouse, CHU de Toulouse, Hôpital Purpan, F-31000, Toulouse, France | Co-investigator |
| Cécile Pays | MD, PhD | Memory Resource and Research Centre of Montpellier, CHU de Montpellier, Hôpital Gui de Chauliac, F-34000, Montpellier, France | Co-investigator |
| Isabelle Pellegrin | MD, PhD | Biological Research Centre, CHU de Bordeaux, F-33000, Bordeaux, France | Co-investigator |
| Rémy Perdrisot | MD, PhD | Memory Resource and Research Centre of Poitiers, CHU de Poitiers, Hôpital de La Milétrie, F-86000, Poitiers, France | Co-investigator |
| Bertille Perin | MD, PhD | Memory Resource and Research of Amiens, CHU Amiens Picardie, F-80000, Amiens, France | Co-investigator |
| Christine Perret-Guillaume | MD, PhD | Memory Resource and Research Centre of Nancy, CHU de Nancy, F-54000, Nancy, France | Co-investigator |
| Grégory Petyt | MD | Memory Resource and Research Centre of Lille, CHRU de Lille, Hôpital Roger Salengro, F-59000, Lille, France | Co-investigator |
| Nathalie Philippi | MD, PhD | Memory Resource and Research Centre of Strasbourg, Hôpitaux Universitaires de Strasbourg, F-67000, Strasbourg, France | Co-investigator |
| Geneviève Pinganaud | MD | Memory Resource and Research Centre of Bordeaux, CHU de Bordeaux, Hôpital Xavier Arnozan, F-33000, Bordeaux, France | Co-investigator |
| Matthieu Plichart | MD | Memory Resource and Research Centre of Paris Broca, AP-HP, Paris, France | Co-investigator |
| Gabriel Pop | MD, PhD | Memory Clinic, Hôpital Avicenne, AP-HP, Hôpitaux Universitaires Paris-Seine-Saint-Denis, F-93009, Bobigny, France | Co-investigator |
| Michèle Puel | MD | Memory Resource and Research Centre of Toulouse, CHU de Toulouse, Hôpital Purpan, F-31000, Toulouse, France | Co-investigator |
| Mathieu Queneau | MD, PhD | Memory Resource and Research Centre of Paris Nord, Centre Cardiologique du Nord, Paris, France | Co-investigator |
| Solène Querellou | MD | Memory Resource and Research Centre of Brest, CHRU de Brest, F-29000, Brest, France | Co-investigator |
| Muriel Quillard-Muraine | MD, PhD | Memory Resource and Research Centre of Rouen, Neurology Department, Rouen University Hospital, F-76031, Rouen, France | Co-investigator |
| Valérie Quipourt | MD, PhD | Memory Resource and Research Centre of Dijon, CHU Dijon Bourgogne, Hôpital du Bocage, Hôpital de Champmaillot, F-21000, Dijon, France | Co-investigator |
| Chloé Rachez | MD, PhD | Memory Resource and Research Centre of Clermont-Ferrand, CHU de Clermont-Ferrand, F-63000, Clermont-Ferrand, France | Co-investigator |
| Micheline Razzouk-Cadet | MD | Memory Resource and Research Centre of Nice, CHU de Nice, Institut Claude Pompidou, F-06100, Nice, France | Co-investigator |
| Anne-Sophie Rigaud | MD, PhD | Memory Resource and Research Centre of Paris Broca, AP-HP, Paris, France | Co-investigator |
| Hélène Robin-Ismer | MD | Memory Resource and Research Centre of Strasbourg, Hôpitaux Universitaires de Strasbourg, F-67000, Strasbourg, France | Co-investigator |
| Mathieu Rodallec | MD, PhD | Memory Resource and Research Centre of Paris Nord, Centre Cardiologique du Nord, Paris, France | Co-investigator |
| Yves Rolland | MD, PhD | Memory Resource and Research Centre of Toulouse, CHU de Toulouse, Hôpital La Grave-Casselardit, F-31000, Toulouse, France | Co-investigator |
| Adeline Rollin-Sillaire | MD, PhD | Memory Resource and Research Centre of Lille, CHRU de Lille, Hôpital Roger Salengro, F-59000, Lille, France | Co-investigator |
| Olivier Rouaud | MD | Memory Resource and Research Centre of Dijon, CHU Dijon Bourgogne, Hôpital du Bocage, Hôpital de Champmaillot, F-21000, Dijon, France | Co-investigator |
| Caroline Roubaud | MD, PhD | Memory Resource and Research Centre of Lyon, Hospices Civils de Lyon, Hôpital des Charpennes, F-69000, Lyon, France | Co-investigator |
| Isabelle Rouch | MD, PhD | Memory Resource and Research Centre of Lyon, Hospices Civils de Lyon, Hôpital des Charpennes, F-69000, Lyon, France | Co-investigator |
| Julie Roux | MD, PhD | Memory Resource and Research Centre of Grenoble, CHU de Grenoble Alpes, Grenoble, France | Co-investigator |
| Guillaume Sacco | MD, PhD | Memory Resource and Research Centre of Nice, CHU de Nice, Institut Claude Pompidou, F-06100, Nice, France | Co-investigator |
| Pierre-Yves Salaun | MD | Memory Resource and Research Centre of Brest, CHRU de Brest, F-29000, Brest, France | Co-investigator |
| François Salmon | MD, PhD | Memory Resource and Research Centre of Poitiers, CHU de Poitiers, Hôpital de La Milétrie, F-86000, Poitiers, France | Co-investigator |
| Alicia Sanchez | MD | Memory Resource and Research Centre of Saint-Etienne, CHU de Saint-Etienne, Hôpital Nord, F-42000, Saint-Etienne, France | Co-investigator |
| Maria-Joao Santiago-Ribeiro | MD, PhD | Memory Resource and Research Centre of Center Region, CHRU de Tours, Hôpital Bretonneau, F-37000, Tours, France | Co-investigator |
| Alain Sarciron | MD | Memory Resource and Research Centre of Lyon, Hospices Civils de Lyon, Hôpital des Charpennes, F-69000, Lyon, France | Co-investigator |
| Nathalie Sastre-Hengan | MD | Memory Resource and Research Centre of Toulouse, CHU de Toulouse, Hôpital La Grave-Casselardit, F-31000, Toulouse, France | Co-investigator |
| Mathilde Sauvée | MD, PhD | Memory Resource and Research Centre of Grenoble, CHU de Grenoble Alpes, Grenoble, France | Co-investigator |
| Christian Scheiber | MD, PhD | Memory Resource and Research Centre of Lyon, Hospices Civils de Lyon, Hôpital des Charpennes, F-69000, Lyon, France | Co-investigator |
| Anne-Marie Schneider | MD, PhD | Memory Resource and Research Centre of Strasbourg, Hôpitaux Universitaires de Strasbourg, F-67000, Strasbourg, France | Co-investigator |
| Franck Semah | MD, PhD | Memory Resource and Research Centre of Lille, CHRU de Lille, Hôpital Roger Salengro, F-59000, Lille, France | Co-investigator |
| Amélie Serra | MD | Memory Resource and Research Centre of Grenoble, CHU de Grenoble Alpes, Grenoble, France | Co-investigator |
| Marie-Laure Seux | MD | Memory Resource and Research Centre of Paris Broca, AP-HP, Paris, France | Co-investigator |
| Hélène Sordet-Guépet | MD | Memory Resource and Research Centre of Dijon, CHU Dijon Bourgogne, Hôpital du Bocage, Hôpital de Champmaillot, F-21000, Dijon, France | Co-investigator |
| Maria Eugenia Soto | MD | Memory Resource and Research Centre of Toulouse, CHU de Toulouse, Hôpital La Grave-Casselardit, F-31000, Toulouse, France | Co-investigator |
| Mathieu Tafani | MD | Memory Resource and Research Centre of Toulouse, CHU de Toulouse, Hôpital Purpan, F-31000, Toulouse, France | Co-investigator |
| Jean-Yves Tanguy | MD, PhD | Memory Resource and Research Centre of Angers, CHU d’Angers, F-49000, Angers | Co-investigator |
| Michael Taroux | MD, PhD | Memory Resource and Research Centre of Dijon, CHU Dijon Bourgogne, Hôpital du Bocage, Hôpital de Champmaillot, F-21000, Dijon, France | Co-investigator |
| Marc Teichmann | MD, PhD | Institute of Memory and Alzheimer's Disease (IM2A), Brain and Spine Institute (ICM), UMR S 1127, Department of Neurology, AP-HP, Pitié-Salpêtrière University Hospital, Sorbonne Universities, Pierre et Marie Curie University, Paris, France | Co-investigator |
| Catherine Terrat | MD, PhD | Memory Resource and Research Centre of Saint-Etienne, CHU de Saint-Etienne, Hôpital de la Charité, F-42000, Saint-Etienne, France | Co-investigator |
| Jamila Thabet | MD | Memory Clinic, Hôpital Avicenne, AP-HP, Hôpitaux Universitaires Paris-Seine-Saint-Denis, F-93009, Bobigny, France | Co-investigator |
| Claire Thalamas | MD | Memory Resource and Research Centre of Toulouse, CHU de Toulouse, Hôpital Purpan, F-31000, Toulouse, France | Co-investigator |
| Catherine Thomas-Anterion | MD, PhD | Memory Resource and Research Centre of Saint-Etienne, CHU de Saint-Etienne, Hôpital Nord, F-42000, Saint-Etienne, France | Co-investigator |
| Anne-Cécile Troussière | MD | Memory Resource and Research Centre of Lille, CHRU de Lille, Hôpital Roger Salengro, F-59000, Lille, France | Co-investigator |
| Renata Ursu | MD | Memory Clinic, Hôpital Avicenne, AP-HP, Hôpitaux Universitaires Paris-Seine-Saint-Denis, F-93009, Bobigny, France | Co-investigator |
| Pierre Vera | MD, PhD | Memory Resource and Research Centre of Rouen, CLCC Henri Becquerel, Rouen, France | Co-investigator |
| Martine Vercelletto | MD | Memory Resource and Research Centre of Nantes, CHU de Nantes, F-44000, Nantes, France | Co-investigator |
| Olivier Vercruysse | MD | Memory Resource and Research Centre of Lille, CHRU de Lille, Hôpital Roger Salengro, F-59000, Lille, France | Co-investigator |
| Antoine Verger | MD, PhD | Memory Resource and Research Centre of Nancy, CHU de Nancy, F-54000, Nancy, France | Co-investigator |
| Philippe Viau | MD | Memory Resource and Research Centre of Nice, CHU de Nice, Institut Claude Pompidou, F-06100, Nice, France | Co-investigator |
| Marie-Neige Videau | MD | Memory Resource and Research Centre of Bordeaux, CHU de Bordeaux, Hôpital Xavier Arnozan, F-33000, Bordeaux, France | Co-investigator |
| Thierry Voisin | MD | Memory Resource and Research Centre of Toulouse, CHU de Toulouse, Hôpital La Grave-Casselardit, F-31000, Toulouse, France | Co-investigator |
| Nathalie Wagemann | MD, PhD | Memory Resource and Research Centre of Nantes, CHU de Nantes, F-44000, Nantes, France | Co-investigator |
| Aziza Waissi-Sediq | MD | Memory Resource and Research Centre of Lyon, Hospices Civils de Lyon, Hôpital des Charpennes, F-69000, Lyon, France | Co-investigator |
| Jing Xie | MD, PhD | Memory Resource and Research Centre of Lyon, Hospices Civils de Lyon, Hôpital des Charpennes, F-69000, Lyon, France | Co-investigator |
| Nathanaëlle Yeni | MD | Laboratoire d'Imagerie Biomédicale, Sorbonne Universités, UPMC Univ Paris 06, Inserm U1146, CNRS UMR 7371, France NeuroSpin, I2BM, Commissariat à l'Energie Atomique, Paris, France | Co-investigator |
| Michel Zanca | MD, PhD | Memory Resource and Research Centre of Montpellier, CHU de Montpellier, Hôpital Gui de Chauliac, F-34000, Montpellier, France | Co-investigator |
| Jean Zinszner | MD, PhD | Memory Clinic, Hôpital Avicenne, AP-HP, Hôpitaux Universitaires Paris-Seine-Saint-Denis, F-93009, Bobigny, France | Co-investigator |
